# The Tyrosine Kinase-Driven Networks of Novel Long Non-coding RNAs and Their Molecular Targets in Myeloproliferative Neoplasms

**DOI:** 10.3389/fcell.2021.643043

**Published:** 2021-08-03

**Authors:** Nonthaphat Kent Wong, Shumeng Luo, Eudora Y. D. Chow, Fei Meng, Adenike Adesanya, Jiahong Sun, Herman M. H. Ma, Wenfei Jin, Wan-Chun Li, Shea Ping Yip, Chien-Ling Huang

**Affiliations:** ^1^Department of Health Technology and Informatics, The Hong Kong Polytechnic University, Kowloon, Hong Kong; ^2^Department of Pathology, United Christian Hospital, Kwun Tong, Hong Kong; ^3^Department of Biology, Southern University of Science and Technology, Shenzhen, China; ^4^Institute of Oral Biology, College of Dentistry, National Yang Ming Chiao Tung University, Taipei, Taiwan

**Keywords:** long non-coding RNAs, myeloproliferative neoplasms, chronic myelogenous leukemia, tyrosine kinase inhibitor, molecular targets

## Abstract

Recent research has focused on the mechanisms by which long non-coding RNAs (lncRNAs) modulate diverse cellular processes such as tumorigenesis. However, the functional characteristics of these non-coding elements in the genome are poorly understood at present. In this study, we have explored several mechanisms that involve the novel lncRNA and microRNA (miRNA) axis participating in modulation of drug response and the tumor microenvironment of myeloproliferative neoplasms (MPNs). We identified novel lncRNAs via mRNA sequencing that was applied to leukemic cell lines derived from *BCR-ABL1*-positive and *JAK2*-mutant MPNs under treatment with therapeutic tyrosine kinase inhibitors (TKI). The expression and sequence of novel LNC000093 were further validated in both leukemic cells and normal primary and pluripotent cells isolated from human blood, including samples from patients with chronic myelogenous leukemia (CML). Downregulation of LNC000093 was validated in TKI-resistant CML while a converse expression pattern was observed in blood cells isolated from TKI-sensitive CML cases. In addition to *BCR-ABL1*-positive CML cells, the driver mutation *JAK2*-V617F-regulated lncRNA BANCR axis was further identified in *BCR-ABL1*-negative MPNs. Further genome-wide validation using MPN patient specimens identified 23 unique copy number variants including the 7 differentially expressed lncRNAs from our database. The newly identified LNC000093 served as a competitive endogenous RNA for miR-675-5p and reversed the imatinib resistance in CML cells through regulating RUNX1 expression. The extrinsic function of LNC000093 in exosomal H19/miR-675-induced modulation for the microenvironment was also determined with significant effect on VEGF expression.

## Introduction

Myeloproliferative neoplasms (MPNs) are characterized by overproduction of blood cells of myeloid lineage stemming from aberrant clonal proliferation of hematopoietic stem cells. Classic MPNs are categorized as Philadelphia chromosome-positive (*BCR-ABL1*-positive; *BCR-ABL1*-pos) or Philadelphia chromosome-negative (*BCR-ABL1*-negative; *BCR-ABL1*-neg). Chronic myelogenous leukemia (CML), being the only *BCR-ABL1*-pos MPN, is caused by a chromosomal translocation that generates the Philadelphia chromosome encoding the constitutively activated tyrosine kinase, *BCR-ABL1* (Deininger et al., 2000; Muxi and Oliver, 2014). *BCR-ABL1*-neg MPNs are further subdivided into several subcategories according to WHO classification, among which the most frequent and distinct subtypes are polycythemia vera (PV), essential thrombocythemia (ET) and primary myelofibrosis (PMF) (Barbui et al., 2018). *BCR-ABL1*-neg MPN patients frequently carry somatic mutations of *Janus kinase 2* (*JAK2*) tyrosine kinase, with *JAK2*-V617F identified as the most prevalent variant that induces overactivation of JAK/STAT signaling and its downstream pathways, which is also observed in MPNs driven by other mutations in *JAK2*, *MPL*, and *CALR* (Pasquier et al., 2014).

Recent research has validated important regulatory roles of non-coding RNAs in leukemogenesis and sensitivity to cancer treatment by drugs (Wang et al., 2016; Chen et al., 2017; Cruz-Miranda et al., 2019). Long non-coding RNAs (lncRNAs) are functionally characterized as a class of regulatory RNAs owing to their potential interactions with various molecules. Among these, H19 and BGL-3 are early identified lncRNAs implicated in the pathogenesis of *BCR-ABL1*-dependent CML (Guo et al., 2014, 2015). H19 is highly expressed in CML in association with hypomethylation of its differentially methylated/imprinted control region (Zhou et al., 2018). Moreover, H19 expression is modulated by the oncogenic transcription factor MYC in leukemic cells (Guo et al., 2014). On the other hand, BGL-3 acts as a tumor suppressor in CML and serves as a competitive endogenous RNA (ceRNA) that competes with microRNA (miRNA) for binding to downstream targets like *PTEN* (Guo et al., 2015). The lncRNA HULC promotes CML cell proliferation by regulating the PI3K/AKT pathway and its expression is positively correlated with clinical stage of CML (Lu et al., 2017). Another lncRNA, HAND2-AS1, is downregulated in CML, and shown to inhibit cell proliferation and enhance apoptosis by removing miRNA-1275 (Yang et al., 2019).

In addition to their involvement in leukemogenesis, lncRNAs modulate the drug sensitivity of CML cells in a *BCR-ABL1*-independent manner. For instance, SNHG5 enhances imatinib resistance by facilitating expression of ABCC2 through binding miR-205-5p (He et al., 2017). Similarly, elevated expression of UCA1 could modulate MDR1 levels by acting as a ceRNA against miR-16 and enhance imatinib resistance via regulation of the drug efflux system (Xiao et al., 2017). Another lncRNA, HOTAIR, is upregulated in CML patients and imatinib-resistant K562 cells with high MRP1 expression. HOTAIR mediates drug resistance via activation of the PI3K/Akt signaling pathway (Wang et al., 2017). On the other hand, the lncRNA MALAT1, a well-known proto-oncogene, enhances the sensitivity of CML cells to imatinib by acting as a miRNA sponge to sequester miRNA-328 (Wen et al., 2018). The lncRNA MEG3 is expressed at extremely low levels in imatinib-resistant CML cells. Notably, its overexpression was reported to reverse imatinib resistance via regulating miR-21 and subsequently influence expression of multidrug resistant transporters (Zhou et al., 2017). The lncRNA NEAT1 regulated by MYC also mediates imatinib-induced apoptosis in CML cells (Zeng et al., 2018). The collective findings suggest that lncRNAs serve as an important regulatory element governing drug resistance in CML patients via collaborative interactions with miRNAs and downstream signaling pathways.

Collectively, evidence has confirmed the involvement of lncRNAs in the JAK/STAT signaling pathway. For instance, SNHG3 acts through the IL6/JAK2/STAT3 pathway to promote proliferation of non-small-cell lung cancer cells (Shi et al., 2020). Another lncRNA, DILC, is also involved in neuropathic pain through regulation of the SOCS3/JAK2/STAT3 pathway (Liu et al., 2020). In melanoma, the lncRNA LHFPL3-AS1 is transcriptionally activated by STAT3 and further enhances STAT3 expression via sponging miR-580-3p to activate JAK2/STAT3 signaling (Peng et al., 2020). Moreover, DLGAP1-AS1 acts as sponge to sequester hepatocellular carcinoma-inhibitory miRNAs, triggering indirect activation of JAK2/STAT3 signaling (Lin et al., 2020). These recent findings support diverse roles of lncRNAs as regulatory components of the JAK/STAT pathway.

While the identification of many novel lncRNAs has led to continuous expansion of the lncRNA database, only very few selected members (<1%) have been functionally characterized and, in particular, none has been reported in *JAK2*-mutant MPNs to date. Indeed, there is no curative treatment for both CML and other MPNs at the moment, but MPNs without effective therapy may progressively transform into severe bone marrow failure or even acute leukemia, while the development of drug resistance is also an obstacle in CML treatment. Hence, it is crucial to improve the existing understanding of disease and treatment mechanisms by investigating novel regulators like lncRNAs, which play an important regulatory role in different processes, and have a high potential to be biological markers in future diagnostic and therapeutic applications. In the present study, we have made considerable efforts to broaden our understanding of the molecular basis of both *BCR-ABL1*-pos and *BCR-ABL1*-neg MPNs. By targeting both known and novel tyrosine kinase-mediated lncRNA-miRNA pathways associated with CML and other MPNs, we evaluated the biological significance of their regulatory functions in drug sensitivity and early hematopoietic gene expression.

## Materials and Methods

### Whole Blood Samples and Nucleic Acid Isolation

Whole blood samples from control subjects, CML patients and *BCR-ABL1*-neg MPN patients were collected and stored with the approval of the Research Ethics Committees of the Hospital Authorities (Hong Kong) and the Hong Kong Polytechnic University including the use of consent forms (Nos. HSEARS20200121004 and HSEARS20160721002). White blood cells were used for nucleic acid isolation following the instructions from the commercial kits, which were used in our local hospitals. RNA samples from ten CML patients were included for LNC000093 detection, and DNA samples from eight PMF patients were included for whole genome sequencing (Supplementary Table 1). Total RNA was extracted with TRIzol^TM^ LS Reagent (Thermo Fisher Scientific) and treated with DNase (Qiagen). Genomic DNA was extracted with the FlexiGene DNA Kit (Qiagen) as per the manufacturer’s instructions. The quality and quantity of extracted RNA and DNA were measured using a NanoDrop 2000 Spectrophotometer (Thermo Fisher Scientific).

### Cell Culture and Clone Selection of Imatinib-Resistant Cells

The human CML cell lines K562, LAMA84-IMS and LAMA84-IMR were purchased from the American Type Culture Collection (ATCC). Cells were grown in RPMI-1640 (Thermo Fisher Scientific) supplemented with 10% fetal bovine serum (FBS; Thermo Fisher Scientific) and incubated at 37°C with 5% CO_2_ in the atmosphere. Imatinib was purchased from Selleckchem (#S2475). The human erythroleukemia (HEL 92.1.7) cell line was a gift from Prof. SP Yip (HKPU, HK) and was cultured in RPMI-1640 medium supplemented with 10% FBS. For JAK2 inhibition assays, 10^5^/mL HEL 92.1.7 cells were treated with 1 μM ruxolitinib (Selleck Chemicals). Human induced pluripotent cells were generated from peripheral blood mononuclear cells (PBMC-iPSCs; ALSTEM). For general maintenance, human PBMC-iPSCs were cultured in Essential 8^TM^ (E8) medium with complete supplements (Thermo Fisher Scientific). Human mesenchymal stromal cells isolated from bone marrow were acquired from Lonza Corporation and maintained in Minimum Essential Medium α (MEMα; no nucleosides, but containing L-glutamine) (Thermo Fisher Scientific) supplemented with 15% FBS (Thermo Fisher Scientific), 5 ng/ml human fibroblast growth factor-basic (hBFGF) (SIGMA) and 1% penicillin/streptomycin.

Imatinib-resistant (IMR) K562 cells (K562-IMR) were generated in our laboratory. In brief, imatinib-sensitive (IMS) K562 cells (K562-IMS) were continuously treated with increasing concentrations of imatinib, starting with 0.5 μM. Cells were subcultured twice a week with an increasing drug concentration (in 0.5 μM increments) following acquisition of survival ability in the presence of imatinib, and stably surviving cell populations under 10 μM imatinib treatment were finally selected. The cell viability assay was performed with 5 × 10^5^ cells/mL seeded in triplicate in 12-well plates and subsequently treated with 3, 5, and 10 μM imatinib for 72 h. After being washed with 1 × phosphate-buffered saline (PBS; Thermo Fisher Scientific), cells were diluted with trypan blue reagent and viability was determined using the Countess II Automated Cell Counter (Thermo Fisher Scientific).

### Poly(A)-Enriched mRNA Sequencing and RT-qPCR

Total RNA was extracted from CML cells with TRIzol^TM^ Reagent (Thermo Fisher Scientific) and the RNeasy Mini Kit (Qiagen), and treated with DNase (Qiagen) to remove contaminating DNA. The RNA integrity number (RIN) was determined using an Agilent 2100 Bioanalyzer (Agilent). An RIN number of >9.5 signifies high quality of isolated RNA. MiRNA was extracted from CML cells with QIAzol^TM^ Reagent (Thermo Fisher Scientific) and an miRNeasy Isolation Kit (Qiagen). The quantity of isolated miRNAs was determined using a Qubit Fluorometer 2.0 (Thermo Fisher Scientific) with the Qubit microRNA assay kit.

Poly(A)-enriched mRNA sequencing (RNA-seq) was performed after library preparation (Groken, Hong Kong). In brief, rRNA was removed using the Ribo-Zero^TM^ magnetic kit, and cDNA library then prepared with the NEBNext^®^ Ultra^TM^ Directional/non-Directional RNA Library Prep Kit for the Illumina HiSeq system.

Reverse transcription (RT) of isolated total RNA was performed with the RevertAid First-Strand cDNA Synthesis Kit (Thermo Fisher Scientific). Quantitative real-time PCR (qPCR) was performed with the QuantiNova SYBR Green PCR Kit (Qiagen). RT of miRNAs was performed using the miScript II RT Kit (Qiagen) and qPCR with the miScript SYBR Green PCR kit (Qiagen). Specific primer pairs were purchased from Integrated DNA Technologies (IDT) (Supplementary Table 2). RT-qPCR procedures were conducted in the standard mode with a ViiA^TM^ 7 Real-Time PCR system (Applied Biosystems).

### Bioinformatics Analysis for Annotating Novel lncRNAs

The RNA-seq raw data quality was assessed with FastQC (Andrews, 2010). Adapters and low-quality bases were removed using Fastp (Chen et al., 2018). After quality control, sequencing reads were mapped using Hisat2 (Kim et al., 2015) to the human reference genome GRCh38. Aligned reads were assembled to known reference and novel transcripts by StringTie (Pertea et al., 2015) with the human reference annotation file as a guide (Cunningham et al., 2019). Novel transcripts were filtered to identify lncRNAs based on the following criteria: (1) transcript length ≥ 200 bp, (2) transcript exon number ≥ 2, and (3) non-coding potential features supported by all four software tools, namely CNCI (Sun et al., 2013), CPAT (Wang et al., 2013), CPC2 (Kang et al., 2017), and PfamScan (Mistry et al., 2007). Differential expression genes (DEGs) were analyzed in DESeq2 (Love et al., 2014). DEGs were annotated in the Metascape database (Zhou et al., 2019) to establish their biological functions. The co-expression network was calculated from DESeq2 normalized gene read counts with a Pearson correlation coefficient threshold > 0.9 and *P*-value < 0.05. The network was visualized and displayed in Cytoscape (Shannon et al., 2003).

### Whole-Genome Sequencing to Identify Recurrent Copy Number Variants in MPN Patients

DNA libraries for whole-genome sequencing were constructed with the MGIEasy FS PCR-Free Library Prep Set (MGI) and amplified products, which were generated by the enzymes included, were obtained after adapter ligation. After heat-denaturation, the 5′ and 3′ terminal adaptors of one strand of the DNA hybridized to a complementary splint oligo to form a nicked circle, which was then ligated to produce a single-stranded DNA circle. The remaining linear molecule was digested with exonuclease for generation of a single-stranded circular DNA library using the DNA-Nanoball technique (MGI). High-throughput sequencing was performed using the MGISEQ-2000 with DNBSEQ-G400RS High-throughput Sequencing Set (FCL PE150).

After barcodes and sequencing adaptors were trimmed, raw sequencing reads were filtered to remove bad reads with low quality and high ‘N’ using the fastp software package. Cleaned sequencing reads in fastq format were mapped to human genome assembly NCBI Build 38 (hg38/NCBI38) using the BWA alignment software with default parameters to generate SAM files. The Genome Analysis Toolkit (GATK 4.1.6.0) was used to convert SAM files to compressed BAM files, sort BAM files by genomic coordinates and remove duplicates with identical start coordinates. The CNVKit package (Talevich et al., 2014) was employed to detect copy number variations. The copy number variations determined were visualized using heatmap, scatter plots and diagram plots, and annotated with genes located within the regions.

### Cell Transfection and Luciferase Reporter Assay

After K562 cells were seeded in six-well-plates at a density of 2 × 10^6^ cells/well, miR-675-3p and -5p mimics (50 nM) were transfected using 5 μl of Lipofectamine 2000 reagent (Invitrogen). The negative control mirVana^TM^ miRNA mimic was transfected in a similar manner into the negative control group. After 24 h of incubation, cultures were replaced with 2 ml fresh medium supplemented with 10% FBS. Cells were harvested at 48 h post-transfection for RNA or protein isolation for use in subsequent assays.

PGL3-CMV-LUC-MCS (Genomeditech, Shanghai, China) expressing firefly luciferase was used to examine the binding capacity of miR-675-5p to target lncRNAs in cells. Firstly, full-length LNC000093 sequences containing the putative wild-type miR-675-5p binding site (5′-UGCACC-3′) and mutant binding site (5′-GUACAA-3′) were synthesized and inserted into the parent vector. After sequence validation by Sanger sequencing, the luciferase vector was amplified and extracted using the EasyPrep HY-Midi Plasmid Extraction Kit (Biotools, Taiwan). K562 cells were further co-transfected with both the luciferase vector and the control plasmid expressing Renilla luciferase in the presence of miR-675-5p mimic, miR-675-3p mimic or negative control miRNA. Cells were harvested and lysed 48 h after transfection and luciferase activity measured using the Dual-Luciferase Reporter Assay System (Promega). The ratio of target Firefly to Renilla luciferase was calculated as relative luciferase activity.

### Protein Extraction and Western Blot Analysis

Total protein was extracted from treated cells using radioimmunoprecipitation assay lysis buffer (50 mM Tris-HCl, pH 7.4, 150 mM NaCl, 1% NP-40) with protease inhibitor and phosphatase inhibitor cocktails. Cells were lysed for 30 min on ice with occasional vortexing and centrifuged at 15,000 *g* for 30 min at 4°C. The amount of extracted protein was quantified with the Pierce^TM^ BCA Protein Assay Kit (Thermo Fisher Scientific) according to the manufacturer’s instructions. In total, 20–40 μg protein was resolved using 8% or 10% Bis-Tris polyacrylamide gels and transferred to polyvinylidene fluoride membrane. The membrane was blocked in PBS with 0.05% Tween-20 and 5% non-fat dry milk, and probed with the appropriate primary antibody overnight at 4°C. After being washed with PBS containing 0.1% Tween 20, the membrane was incubated with horseradish peroxidase-conjugated secondary antibodies at room temperature for 1 h. Signals were visualized with an enhanced chemiluminescence detection system. Antibody information is provided in Supplementary Table 3.

### CRISPR-Cas9 Genome Editing

To generate plasmid-based single-guide RNA (sgRNA) constructs (Supplementary Table 4), the all-in-one CRISPR-Cas9 vector (pAll-Cas9.Ppuro; Academia Sinica, Taiwan) was used as the backbone for cloning. Transfection was performed with the Neon Transfection Kit (Thermo Fisher Scientific). In total, 3 × 10^5^ K562 cells were collected from each culture flask, washed twice with 1 × PBS, centrifuged at 200 *g* for 5 min, and incubated in 10 μl resuspension Buffer R. Viability of K562-edited cells under different electroporation conditions was examined at 1 and 7 days after electroporation with the trypan blue exclusion method. DNA extraction and H19 CRISPR cleavage detection via PCR and gel electrophoresis were further performed after puromycin selection.

For transfection into human PBMC-iPSCs, the Alt-R CRISPR-Cas9 system (IDT) was used to generate a LNC000093-deleted allele. A pair of custom sgRNAs targeting two genomic regions flanking LNC000093 (Supplementary Table 4) was designed with proprietary algorithms of IDT. The sgRNAs were mixed with Alt-R^®^ S.p. Cas9 Nuclease V3 (IDT) to form ribonucleoprotein (RNP) complexes, which were subsequently used to assemble transfection complexes by incubation with Lipofectamine RNAiMAX reagent (Invitrogen). In total, 3 × 10^5^ cells per well in 24-well plates were transfected with 10 nM RNP complex.

### Validation of the LNC000093 Sequence

Full-length LNC000093 was amplified from cDNA samples and cloned using the TOPO^TM^ XL-2 Complete PCR Cloning Kit (Invitrogen). Selected clones were sequenced using an ABI 3130 Genetic Analyzer Sequencer with the BigDye^TM^ Terminator v1.1 Cycle kit (Applied Biosystems^TM^).

### Embryoid Body Formation for Human PBMC-iPSC Differentiation

For embryoid body (EB) formation, Essential 6^TM^/Polyvinyl Alcohol Medium (E6/PVA) was used to support spontaneous differentiation of human iPSCs using the Spin EB formation method. In brief, E8 medium from human iPSC cell cultures was aspirated and wells rinsed once with PBS. Next, 0.5 mL ReLeSR (STEMCELL Technologies) per well was added and aspirated within 1 min to expose iPSC colonies to a thin film of ReLeSR liquid. The Geltrex-coated 6-well plate was incubated at room temperature for 7–9 min. After incubation, 1 mL per well of prepared E6/PVA medium with 10 μM ROCK inhibitor (Y-27632) (STEMCELL Technologies) was gently added to the wells and colonies detached by tapping the side of the plate for 30–60 s. iPSC aggregates were dissociated into single cells by means of pipetting up and down using 1-mL autopipettes. Subsequently, iPSCs were counted and diluted to the appropriate cell densities using E6/PVA with 10 μM ROCK inhibitor (Y-27632). Next, iPSCs were seeded at 8,000 cells/well in 100 μL medium (equivalent to 80,000 cells/mL) in a 96-well plate. Next, the cell suspension (100 μL) was pipetted into each well of the 96-well plate. The plate was centrifuged at 300 *g* for 5 min and subsequently incubated at 37°C with 5% CO_2_ overnight.

For directed hematopoietic differentiation, embryoid bodies were formed as mentioned above for 4 days and transferred to a six-well plate with StemPro^TM^-34 SFM (Gibco^TM^) supplemented with 2 mM glutamine, 50 μg/mL ascorbic acid, 150 μg/mL transferrin, 0.4 mM monothioglycerol and various cytokines as follows: days 4–9: 30 ng/mL BMP4, 50 ng/mL VEGF, 50 ng/mL SCF, 50 ng/mL TPO, 50 ng/mL Flt3L, and 20 ng/mL bFGF (PeproTech); days 9–14: 30 ng/mL BMP4, 50 ng/mL SCF, 50 ng/mL TPO, 10 ng/mL IL-3, 5 ng/mL IL-11, 2 U/mL EPO, and 125 ng/mL IGF.

### Exosome Isolation and Flow Cytometry Analysis

Exosomes were isolated with ExoQuick-TCTM Exosome Precipitation Solution (System Biosciences). In brief, culture medium was collected via centrifugation at 3,000 *g* for 15 min to remove cells and cell debris. Next, 50 mL supernatant was transferred and mixed with 10 mL ExoQuick-TC Exosome Precipitation Solution. After overnight (16 h) incubation, exosomes were collected via centrifugation at 1,500 *g* for 60 min and subjected to RNA or protein extraction.

For analysis of exosome enrichment, suspended exosomes were incubated with CD63 antibody-conjugated Dynabeads (Thermo Fisher Scientific); CD63 is often used as a marker for exosomes. After magnet-based enrichment reactions, bead-bound exosomes were subjected to CD63-PE antibody staining, followed by washing steps with PBS. Flow cytometry analysis was performed using CellQuest software (BD Biosciences).

### Statistical Analysis

GraphPad Prism (version 7.0; GraphPad Software, San Diego, CA, United States) was used to perform all statistical analyses. All data are presented as mean ± SD values from at least three independent biological replicates unless otherwise stated. Unpaired Student’s *t*-test was used for statistical analysis between two groups and one-way ANOVA for analysis involving more than two groups. *P*-value < 0.5 was considered to be statistically significant. Statistical significance was indicated as ^∗^*P* < 0.05 or ^∗∗^*P* < 0.01.

## Results

### Identification of Novel Non-coding RNA Pathways in *BCR-ABL1*-Positive CML and -Negative MPN-Derived Cells

#### Upregulation of H19 and miR-675 in Imatinib-Resistant CML Cells

**FIGURE 1:**
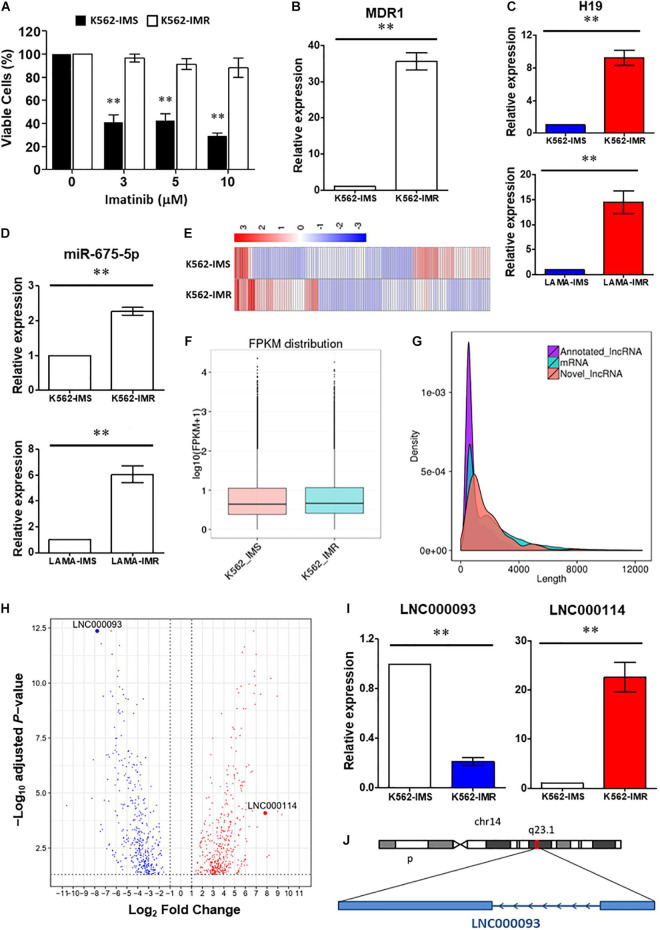
Identification of lncRNAs involved in imatinib-resistant CML cells. **(A)** Cell viability of K562-IMS and K562-IMR upon imatinib treatment with different dosage was determined by trypan blue exclusion assay (*n* = 3). The mean ± SD is shown for the results. ^∗∗^*P* < 0.01. **(B)** RT-qPCR analysis revealed a significant upregulation of MDR1 expression in K562-IMR relative to K562-IMS (*n* = 6). The mean ± SD is shown for the results. ^∗∗^*P* < 0.01. **(C,D)** The relative expression changes of H19 **(C)** and miR-675-5p **(D)** in K562 and LAMA84 were analyzed by RT-qPCR (*n* = 4–6). The mean ± SD is shown for the results. ^∗∗^*P* < 0.01. **(E)** The Heat map reflected differentially expressed genes in RNA-seq for K562-IMS and K562-IMR. **(F)** The box plot showed the FPKM distribution of K562-IMS and K562-IMR samples in RNA-seq. **(G)** The density plot showed the length difference of lncRNAs and mRNAs. **(H)** The volcano plot showed differentially expressed genes of K562-IMR relative to K562-IMS. The horizontal dotted line was the threshold of adjusted *P*-value < 0.05. The red dots represent upregulated genes, and blue dots represent downregulated genes. The most significant downregulated novel lncRNA LNC000093 and upregulated novel lncRNA LNC000114 were highlighted. **(I)** RT-qPCR analysis revealed a significant downregulation of LNC000093 and upregulation of LNC000114 in K562-IMR cells relative to K562-IMS cells (*n* = 3–6). The mean ± SD is shown for the results. ^∗∗^*P* < 0.01. **(J)** The novel lncRNA LNC000093 is located on chromosome 14q23.1 and is transcribed in anti-sense direction with 2 exons.

Accumulating studies have reported a major oncogenic role of the lncRNA H19 (Yoshimura et al., 2018; Ghafouri-Fard et al., 2020). This lncRNA produces a 2.3 kb functional transcript and serves as a reservoir for miR-675. In keeping with the reported functions of miRNAs, miR-675 exerts negative regulatory effects on specific targets. To investigate the detailed molecular pathway of H19/miR-675 activity in CML drug resistance, K562 and LAMA84 cells were selected under TKI treatment. Imatinib-resistant K562 cells (K562-IMR) showed >88.1% viability, which was markedly higher than that of imatinib-sensitive K562 cells (K562-IMS) that showed <28.7% viability after 72 h of treatment (10 μM) (Figure 1A). In addition, 35.6-fold upregulation of MDR1/P-gp gene expression was detected in K562-IMR cells (Figure 1B).

To determine the involvement of H19 in imatinib resistance, RT-qPCR was performed to compare H19 expression patterns between K562-IMR and K562-IMS cells. H19 expression was significantly upregulated by 9.2-fold in K562-IMR cells when compared with K562-IMS cells (Figure 1C upper panel; *P* < 0.01). Similar results were obtained with the CML LAMA84 cell line, with a 15.1-fold increase in H19 in LAMA84-IMR cells (Figure 1C lower panel; *P* < 0.01). Consistent with H19 expression, upregulation of mature miR-675-5p was detected in both K562- and LAMA84-IMR cells (2.3- and 6.1-fold increase, respectively; Figure 1D). On the other hand, converse regulation patterns of H19/miR-675-5p in imatinib-sensitive cells were detected upon imatinib treatment in a dose-dependent manner while changes in miR-675-3p upon treatment were not significant (Supplementary Figure 1).

To further identify novel regulators in the modulation of imatinib resistance, poly(A)-enriched RNA-seq was performed to target differentially expressed lncRNAs, including novel transcripts. Differential expression analysis revealed the presence of genes with significant differences between IMR and IMS cells. Clustering analysis based on hierarchical clustering was performed with FPKMs (expected number of Fragments Per Kilobase of transcript sequence per Million base-pairs sequenced) using log_10_(FPKM + 1) for clustering. Red represents genes with higher expression in IMR cells than in IMS cells while blue represents those with lower expression in IMR cells than in IMS cells (Figure 1E). Significantly expressed lncRNAs were considered in cluster construction. In total, 144 differentially expressed lncRNAs were identified, among which 122 were annotated lncRNAs (with known sequences) and 22 were novel lncRNAs (sequences newly identified from our data that have not been published). The procedure of bioinformatics analysis for annotating novel lncRNAs is depicted (Supplementary Figure 2) and their differential expression with length differences further validated (Figures 1F,G).

#### LNC000093 Is a Novel Downregulated lncRNA in Imatinib-Resistant CML Cells

Differentially expressed genes and lncRNAs were obtained by comparing K562-IMR to -IMS cells via RNA-seq. The log_2_ fold-changes (log_2_FC) were analyzed from biological replicates with correction for batch effect (Figure 1H). After functional annotation through Gene Ontology and KEGG pathways, a few relevant top associated pathways were identified as cytokine-mediated signaling, cell morphogenesis involved in differentiation and the tyrosine kinase pathway (Supplementary Figure 3). Interestingly, numerous differentially expressed genes were associated with blood vessel development, exocytosis and regulation of secretion (Supplementary Figure 3), and this suggests that it may be worth exploring functional studies on extrinsic regulatory mechanisms.

Among the newly annotated lncRNAs identified by our group, we used one of the most significantly downregulated lncRNAs (LNC000093; log_2_FC = −7.77155, adjust *P*-value = 4.31E-13; Figure 1H) in RNA-seq as the target to examine the potential post-transcriptional inhibitory effects of H19/miR-675-5p. RT-qPCR results confirmed significant downregulation of LNC000093 in imatinib-resistant CML cells (Figure 1I; *P* < 0.01) and, conversely, upregulation of H19 (Figure 1C; *P* < 0.01) and another novel lncRNA, LNC000114 (Figure 1I; *P* < 0.01). The expression changes of LNC000093 and LNC000114 in K562-IMR relative to K562-IMS, detected via RT-qPCR, showed similar trend to those determined with RNA-seq analysis (Figure 1H).

**FIGURE 2:**
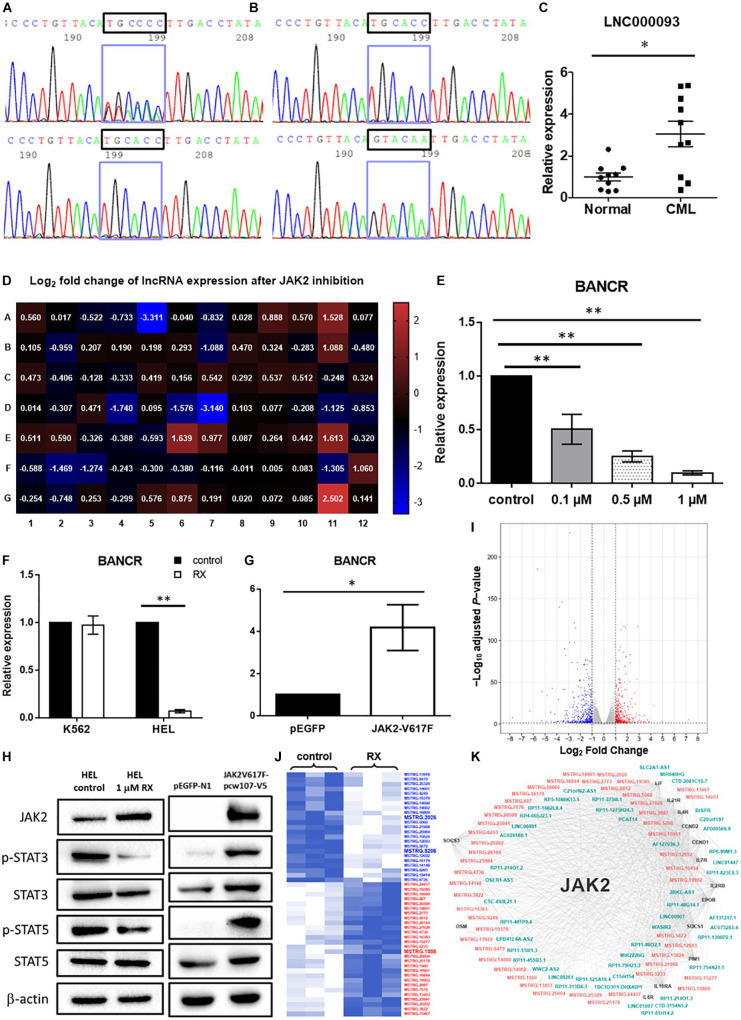
Validation of novel lncRNAs in CML patients and MPN-like cells upon TKI treatment. **(A)** Cycle sequencing analysis using cDNA as template revealed 2 variants of 6 continuous nucleotide polymorphisms in exon 2 of LNC000093 **(upper panel)**, while only one variant was shown when using genomic DNA as template **(lower panel)**. **(B)** Amplicons from cDNA template were cloned into empty vector and two isolated variants (TGCACC/GTACAA) were selected and evaluated by cycle sequencing. **(C)** RT-qPCR analysis detected an upregulated expression of LNC000093 in white cells from TKI-sensitive CML patients (*n* = 10) relative to healthy controls (*n* = 10). The mean ± SD is shown for the results. ^∗^*P* < 0.05. **(D)** Real-time qPCR array assay revealed expression of 84 tumorigenesis-related lncRNA in HEL 92.1.7 treated with ruxolitinib for 48 h. **(E)** The expression level of BANCR in HEL 92.1.7 cells treated with different ruxolitinib concentrations was detected by RT-qPCR (*n* = 4). The mean ± SD is shown for the results. ^∗∗^*P* < 0.01. **(F)** The expression change of BANCR upon ruxolitinib treatment in HEL 92.1.7 and K562 cells was analyzed by RT-qPCR (*n* = 3). The mean ± SD is shown for the results. ^∗∗^*P* < 0.01. **(G)** RT-qPCR analysis revealed an upregulation of BANCR expression in HEK293T cells transfected with *JAK2*-V617F overexpressing vector (*n* = 6). The mean ± SD is shown for the results. ^∗^*P* < 0.05. **(H)** Western blots showed a reduced activation of STAT3 and STAT5 phosphorylation in ruxolitinib-treated HEL 92.1.7 **(left panel)** but strong activation of STAT3 and STAT5 phosphorylation in HEK293T overexpressing *JAK2*-V617F. **(I)** The volcano plot showed differentially expressed genes (adjusted *P*-value < 0.05 and | fold change| ≥ 2) upon ruxolitinib treatment in HEL 92.1.7. The red dots represent upregulated genes, and blue dots represent downregulated genes. **(J)** The heat map reflected novel lncRNAs with differential expression (≥2.0-fold) in RNA-seq identified after the treatment of JAK2 inhibitor – ruxolitinib (1 μM) for 48 h. LncRNAs labeled blue showed downregulation, while red labeling showed upregulation. **(K)** The gene co-expression network represented associations among differentially expressed JAK2-pathway genes (black), known lncRNAs (blue–green) and novel lncRNAs (orange–pink).

Sequence analysis of the full-length LNC000093 transcript (1,418 bp; mapping to the genome region located on chromosome 14q23.1; Figure 1J) was performed in CML cell lines (K562 and LAMA84), human PBMCs and human iPSCs. Interestingly, in addition to known nucleotides with reported polymorphisms in the SNP database (NCBI), we identified a new polymorphism containing six continuous nucleotides in exon 2 of LNC000093 (Figures 2A,B). Surprisingly, the two alleles were detected in RNA transcripts (Figure 2A, upper panel), but not genomic DNA (Figure 2A, lower panel), with cycle sequencing. The two variants (TGCACC/GTACAA) were further verified in isolated single clones of plasmids (Figure 2B). Moreover, the expression of LNC000093 was upregulated in the white cells from TKI-sensitive CML patients (Figure 2C) and, conversely, downregulated in CML-IMR cells (Figure 1I and Supplementary Figure 4A).

#### Diverse lncRNA Expression Patterns in *BCR-ABL1*-neg MPN With *JAK2*-V617F Driver Mutations

To screen for potential lncRNA candidates involved in *JAK2*-V617F signaling, a prevalent driver mutation of *BCR-ABL1*-neg MPNs, 84 tumorigenesis-related lncRNAs were assessed via RT-qPCR array in both control and ruxolitinib-treated HEL 92.1.7 cells (48 h post treatment) (Figure 2D); ruxolitinib is a JAK inhibitor. LncRNAs showing the most significant differences in expression in response to JAK2 inhibition were potential targets (BANCR, CBR3-AS1, LINC00261, LINC00887, LUCAT1, and NBR2) for further investigation (Table 1). Among these, H19 was upregulated after JAK2 inhibition (Supplementary Figure 4B), opposite to *BCR-ABL1* inhibition in CML cells (Supplementary Figure 1A). The lncRNA displaying the most significant difference, BANCR, was selected for further investigation of its involvement in *JAK2*-V617F induction. The full name for BANCR is BRAF-activated non-protein coding RNA (Table 1).

**TABLE 1:** LncRNAs showing significant differential expression after JAK2 inhibition by ruxolitinib treatment.

Position*	Gene symbol	Fold change	Log_2_ fold change^#^	*t*-test *P-*value^†^	Description
A05	BANCR	0.101	−3.308	0.0051	BRAF-activated non-protein coding RNA
A09	CBR3-AS1	1.851	0.888	0.0043	CBR3 antisense RNA 1
D04	LINC00261	0.299	−1.742	0.0105	Long intergenic non-protein coding RNA 261
D07	LINC00887	0.113	−3.146	0.00046	Hypothetical LOC100131551
D12	LUCAT1	0.554	−0.852	0.0016	Lung cancer associated transcript 1 (non-protein coding)
E09	NBR2	1.201	0.264	0.0066	Neighbor of BRCA1 gene 2 (non-protein coding)

*This refers to the position shown in Figure 2D (as one representative result). ^#^The values are calculated as the average from three biological replicates. ^†^The P-values are calculated using a Student’s t-test (two-tail distribution and equal variances between the two samples) on the replicate 2^–ΔCT^ values for each gene in the treatment group compared to the control group.

Dose-dependent downregulation of BANCR was observed in HEL 92.1.7 cells after ruxolitinib treatment (0.1, 0.5, and 1 μM) at 48 h (Figure 2E). BANCR expression was inhibited in two *JAK2*-V617F^+^ cell lines, namely HEL 92.1.7 and SET-2, by 10.2- and 6.94-fold, respectively (Figure 2F and Supplementary Figure 4C; *P* < 0.01), but not *JAK2* wild-type K562 cells (Figure 2F). In contrast, exogenous expression of *JAK2*-V617F in HEK293T cells induced 4.18-fold upregulation of BANCR when compared to vector-control transfected cells (Figure 2G; *P* < 0.05). These results demonstrate direct regulation of BANCR expression by *JAK2*-V617F as well as downstream JAK/STAT signaling activation as determined from STAT3 and STAT5 phosphorylation (Figure 2H).

### Molecular Signaling and Interactions of Novel lncRNA Pathways

#### Novel *JAK2*-Associated lncRNA Networks and Their Putative Interactions With Competing BANCR-MicroRNA-Mediated JAK/STAT Pathways

To identify the novel *JAK2*-associated lncRNA networks, large-scale screening of control and ruxolitinib-treated (48 h) HEL 92.1.7 cells was conducted using RNA-seq. In total, 908 differentially expressed genes were identified from three biological replicates analyzed with correction for batch effect (Figure 2I), including 51 predicted novel lncRNAs (Figure 2J). In differential expression analysis, co-expression network analysis of JAK2-pathway genes and lncRNAs revealed plausible interrelationships, consistent with the finding that JAK2 plays a central regulatory role in differential expression of lncRNAs (Figure 2K and Supplementary Table 5).

In addition to expression changes as the initial criteria for determining the potential significance of newly identified lncRNAs, their locations in the genome were also analyzed for mapping with clinically significant copy number variations (CNVs) in MPN patients. Interestingly, 7 differentially expressed novel lncRNAs from our database were mapped to regions of MPN-related CNVs according to our and other CNV analyses using whole-genome sequencing in MPN patients (Supplementary Table 6). Among these, MSTRG.1558, one of the top 20-upregulated novel lncRNAs in HEL 92.1.7 subjected to ruxolitinib treatment (Supplementary Table 5), is located within the region chr1: 124804374–124817081 that contains an MPN-related CNV with a lower copy number. Conversely, MSTRG.2026 was identified as one of the top 20-downregulated novel lncRNAs (Supplementary Table 5) located within the region chr1: 161450633–161458545 containing 4 copies of an MPN-related CNV (Supplementary Table 6). Copy number and expression of MSTRG.2026 were positively correlated with the activation of JAK2 signaling.

The interaction networks of BANCR-miRNA-novel lncRNAs were further analyzed to determine putative miRNA interactions between BANCR and other *JAK2*-mediated lncRNAs that could support their potential competing regulatory role as ceRNAs. We targeted a miRNA, miR-3609, which has been shown to be upregulated after JAK2 inhibition (Pagano et al., 2018). Following *in silico* analysis with STarMir (Rennie et al., 2014), putative binding regions with a matching seed sequence (AGUGAAA) were identified in BANCR and 8 other novel lncRNAs, which were differentially downregulated from our RNA-seq data (analyzed from the top 20 with highest fold-change; Supplementary Tables 5, 7). Among the top 20 novel downregulated lncRNAs, MSTRG.5206 showed the most significant changes (log_2_FC = −5.27; Supplementary Table 5). Moreover, miR-3609 potentially interacts with 10 genes involved in JAK/STAT signaling (Supplementary Table 8). Further experimental follow-up by means of whole transcriptome analysis in MPN patients will be required to prove the putative interaction networks obtained with bioinformatics analysis.

#### LNC000093 Expression Is Regulated by H19/miR-675-5p via Direct Binding

Among the significantly downregulated novel lncRNAs in IMR cells (top 20), only LNC000093 contained three binding regions (BR) for miR-675-5p, as determined by *in silico* analysis. The three putative binding regions were near the 3′ end of LNC000093 transcripts, designated BR1 [971–1,008], BR2 [1,237–1,268], and BR3 [1,302–1,322], with perfect matching to the seed sequence (GGUGCG) of miR-675-5p (Supplementary Figure 5A).

**FIGURE 3:**
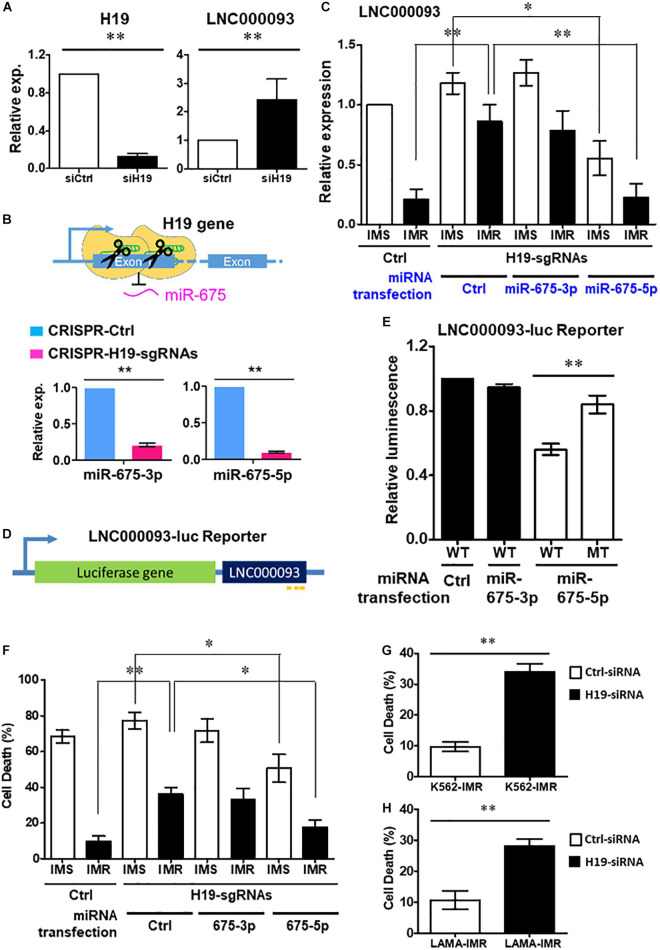
Regulation of LNC000093 expression by H19/miR-675-5p via direct binding sequence. **(A)** An increased expression of LNC000093 was detected in K562 cells transfected with H19-siRNA (*n* = 3). The mean ± SD is shown for the results. ^∗∗^*P* < 0.01. **(B)** After CRISPR-Cas9-mediated deletion of H19/miR-675, both miR-675-3p and −5p showed significant downregulation as shown by RT-qPCR analysis (*n* = 3). The mean ± SD is shown for the results. ^∗∗^*P* < 0.01. **(C)** RT-qPCR analysis revealed an enhanced LNC000093 expression in H19/miR-675-deleted K562-IMR cells. Overexpression of miR-675-5p via microRNA transfection reversed the upregulation of LNC000093 in H19/miR-675-deleted K562-IMR and induced a downregulation of LNC000093 in H19/miR-675-deleted K562-IMS (*n* = 3). The mean ± SD is shown for the results. ^∗^*P* < 0.05, ^∗∗^*P* < 0.01. **(D)** A luciferase reporter vector was constructed with insertion of the full-length LNC000093 cDNA sequence into the 3′ end of luciferase gene. **(E)** A reduction of the luciferase activity was detected in miR-675-5p and BR1-3-WT co-transfected cells, as compared with BR2-MT co-transfected cells (*n* = 3). The mean ± SD is shown for the results. ^∗∗^*P* < 0.01. **(F)** The cell death percentage of K562-IMR under imatinib treatment was enhanced by the CRISPR-Cas9-mediated H19/miR-675 deletion and was reversed by miR-675-5p overexpression (*n* = 3). The mean ± SD is shown for the results. ^∗^*P* < 0.05, ^∗∗^*P* < 0.01. **(G)** The cell death percentage of K562-IMR under imatinib treatment was enhanced by the siRNA-mediated H19 inhibition (*n* = 3). The mean ± SD is shown for the results. ^∗∗^*P* < 0.01. **(H)** The cell death percentage of LAMA84-IMR under imatinib treatment was enhanced by the siRNA-mediated H19 inhibition (*n* = 3). The mean ± SD is shown for the results. ^∗∗^*P* < 0.01.

To verify the regulatory effects of H19 on LNC000093, loss-of-function studies using H19 small interfering RNA (H19-siRNA) and CRISPR-Cas9-mediated deletion were performed. Following knockdown of H19 expression (down to 0.13-fold), increased expression of LNC000093 was detected (2.6-fold) in H19-siRNA transfected K562 cells (Figure 3A; *P* < 0.01). To further determine whether this effect is attributable to H19-derived miR-675-3p or miR-675-5p, we generated H19-deleted cells using the CRISPR-Cas9 system. Specific deletion of the miR-675 sequence was achieved via cleavage with two H19 single-guide RNAs (H19-sgRNA; Supplementary Table 4), and subsequent downregulation of both miR-675-3p and -5p confirmed via RT-qPCR (Figure 3B; *P* < 0.01). H19/miR-675-deleted K562-IMR cells exhibited a 4.1-fold increase in LNC000093 expression (Figure 3C; *P* < 0.01), which was reversed under conditions of overexpression of miR-675-5p (Figure 3C; *P* < 0.01), but not miR-675-3p via transfection with the respective miR-675 species. Moreover, transfection of miR-675-5p into K562-IMS cells led to 2.1-fold downregulation of LNC000093 (Figure 3C; *P* < 0.05).

In addition, we constructed a luciferase reporter vector with insertion of full-length LNC000093 cDNA sequence at the 3’ end of the luciferase gene (Figure 3D) to evaluate whether miR-675-5p exerted its effects through direct interaction with LNC000093. LNC000093-reporter vectors containing either the miR-675-5p wild-type binding sequences (BR1-3-WT) or mutant binding sequence (BR2-MT; with GUUCAA replacing UGCACC in BR2) of LNC000093 were generated. Overexpression of miR-675-5p reduced the luciferase activities of BR1-3-WT by 48% relative to BR2-MT (Figure 3E; *P* < 0.01), while transfection of control miRNA or miR-675-3p did not cause significant decrease in luciferase activities of BR1-3-WT. Our collective findings clearly indicate that LNC000093 expression is regulated by H19/miR-675-5p via a process involving direct binding.

### Intrinsic and Extrinsic Functions of the Identified Novel lncRNA Pathways

#### MiR-675-5p-Suppressed Cell Death and RUNX1 Expression in Imatinib-Resistant Cells Are Rescued by LNC000093

**FIGURE 4:**
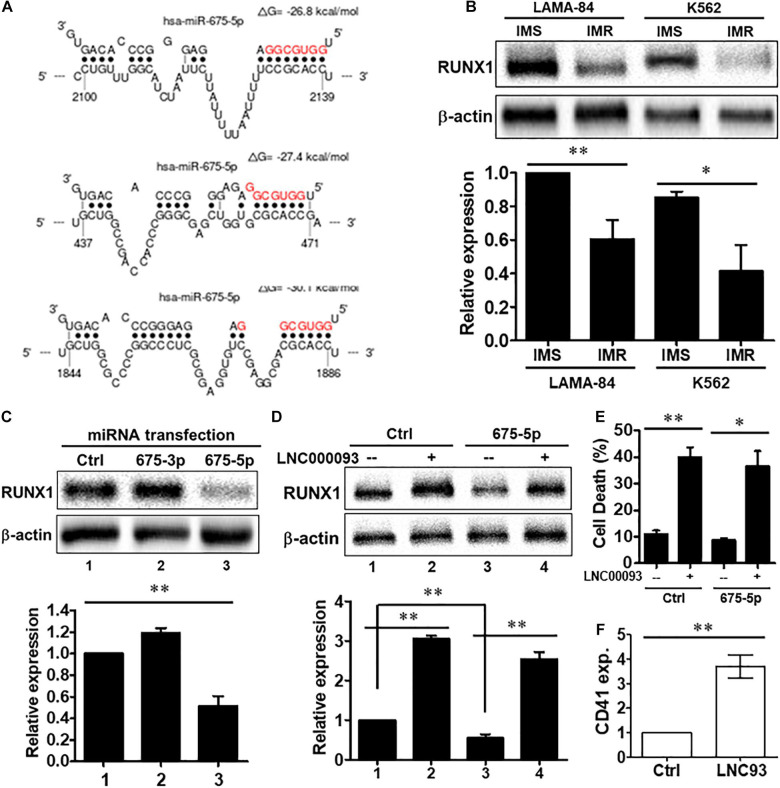
miR-675-5p-suppressed cell death and RUNX1 expression in imatinib-resistant cells were rescued by LNC000093 expression. **(A)** *In silico* analysis revealed three putative binding sites of miR-675-5p on RUNX1. **(B)** Western blots showed a downregulation of RUNX1 in K562-IMR and LAMA84-IMR (*n* = 3). The mean ± SD is shown for the results. ^∗^*P* < 0.05, ^∗∗^*P* < 0.01. **(C)** A downregulation of RUNX1 in K562 cells transfected with miR-675-5p mimics was revealed by Western blots (*n* = 3). The mean ± SD is shown for the results. ^∗∗^*P* < 0.01. **(D)** Western blots showed an enhanced expression of RUNX1 in cells overexpressing LNC000093. The reduced RUNX1 expression induced by miR-675-5p was rescued by ectopic expression of LNC000093 (*n* = 3). The mean ± SD is shown for the results. ^∗∗^*P* < 0.01. **(E)** The cell death percentage of K562-IMR upon imatinib treatment was increased by LNC000093 overexpression with or without co-transfection of miR-675-5p mimics (*n* = 3). The mean ± SD is shown for the results. ^∗^*P* < 0.05, ^∗∗^*P* < 0.01. **(F)** RT-qPCR analysis showed an enhanced CD41 expression in LNC000093-overexpressing K562 with 48-h PMA treatment (*n* = 3). The mean ± SD is shown for the results. ^∗∗^*P* < 0.01.

To determine the potential involvement of LNC000093 in imatinib-induced cell death, we examined the effects of H19/miR-675-5p manipulation on K562-IMS and -IMR cells. Inhibition of miR-675 via CRISPR-Cas9 enhanced cell death 3.6-fold (Figure 3F; *P* < 0.01) after imatinib treatment (10 μM) of K562-IMR cells for 72 h. This finding was validated using H19 siRNA, which induced a similar 3.5-fold increase in K562-IMR cell death and 2.8-fold increase in LAMA84-IMR cell death (Figures 3G,H; *P* < 0.01). This increase in cell death was reversed by overexpression of miR-675-5p, but not by miR-675-3p overexpression (Figure 3F; *P* < 0.05). The molecular target of miR-675-5p, RUNX1, an important hematopoietic transcription factor containing three putative miR-675-5p binding sites (Figure 4A), was downregulated in both LAMA84- and K562-IMR cells (Figure 4B; *P* < 0.01 and 0.05, respectively). The inhibitory effect on RUNX1 protein expression was exerted through miR-675-5p overexpression in K562 cells, as demonstrated with miR-675-5p mimics (Figure 4C; *P* < 0.01). The collective findings suggest that the post-transcriptional regulatory pathway driven by miR-675-5p functionally inhibits expression of wild-type RUNX1, subsequently reducing drug sensitivity to imatinib, which is reflected by the percentage of IMR cell death (Figures 3F, 4B,C).

We further focused on the putative role of LNC000093 as a ceRNA for miR-675-5p and consequent de-repression of RUNX1 expression. To examine our hypothesis, co-expression of LNC000093 and miR-675-5p was performed using the pCMV expression plasmid and synthetic miRNA mimic. RUNX1 expression was enhanced 4.6-fold upon overexpression of both LNC000093 and miR-675-5p (Figure 4D; *P* < 0.01), suggesting that miR-675-5p-induced inhibition of RUNX1 expression is rescued by ectopic expression of LNC000093. Increased RUNX1 expression (3.1-fold) was also observed in the miRNA control group following LNC000093 overexpression (Figure 4D; *P* < 0.01).

In addition to regulation of gene expression, LNC000093 also affects cellular drug sensitivity and differentiation markers. Forced expression of LNC000093 led to increased cell death of K562-IMR (from 11.1 to 40.0% in the control group and 8.7–36.6% in the miR-675-5p transfected group; Figure 4E; *P* < 0.01 and 0.05, respectively). Regarding its effect on differentiation state, K562 cells were induced with phorbol-myristate-acetate (PMA) to megakaryocytic lineage, in which RUNX1 is reported to play a regulatory role (Elagib et al., 2003; Pencovich et al., 2011). After 48 h treatment with PMA, the megakaryocytic marker, CD41, was upregulated 3.7-fold in LNC000093-overexpressing relative to vector-control transfected cells (Figure 4F; *P* < 0.01). Our results demonstrate for the first time that LNC000093 acts as a ceRNA to regulate cell viability in response to TKI treatment through competing with H19/miR-675-5p-mediated RUNX1 inhibition.

#### LNC000093 Is Required to Sustain Expression of CD34, CXCR4, and GATA2 During Early Hematopoietic Differentiation of Human Induced Pluripotent Stem Cells

**FIGURE 5:**
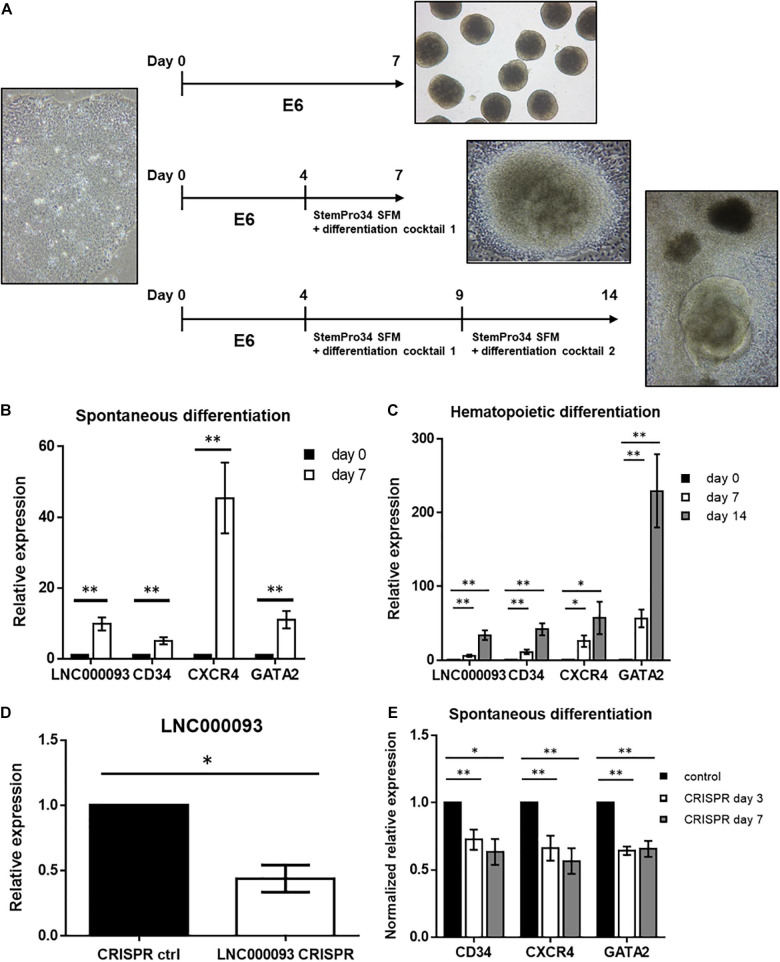
Potential role of LNC000093 in hematopoietic differentiation. **(A)** A schematic diagram of the culture protocol for three different iPSC differentiation schedules. **(B,C)** RT-qPCR analysis showed upregulation of LNC000093, CD34, CXCR4, and GATA2 expression upon **(B)** 7-day spontaneous differentiation (*n* = 4), and **(C)** 7- and 14-day hematopoietic differentiation (*n* = 6). The mean ± SEM is shown for the results. ^∗^*P* < 0.05, ^∗∗^*P* < 0.01. **(D)** The expression level of LNC000093 after CRISPR-deletion in iPSC was evaluated by RT-qPCR (*n* = 3). The mean ± SEM is shown for the results. ^∗^*P* < 0.05. **(E)** RT-qPCR analysis revealed a reduced relative expression change of CD34, CXCR4, and GATA2 in 3-day and 7-day LNC000093-CRISPR-deleted EB with normalization to control EB (*n* = 6). The mean ± SEM is shown for the results. ^∗^*P* < 0.05, ^∗∗^*P* < 0.01.

To further establish the functional role of LNC000093 during hematopoietic differentiation, the human PBMC-iPSC system coupled with CRISPR-Cas9-mediated deletion of LNC000093 was employed. Spontaneous and hematopoietic differentiation were conducted after EB formation (Figure 5A). On day 7 of spontaneous differentiation (first row of Figure 5A), LNC000093 and the hematopoietic lineage markers (CD34, CXCR4, and GATA2) were upregulated by 9.82-fold (LNC000093), 5.06-fold (CD34), 45.44-fold, (CXCR4), and 11.02-fold (GATA2), respectively (Figure 5B; *P* < 0.01). Similar trends were obtained after directed hematopoietic differentiation using cytokine cocktails for 7 and 14 days (Figure 5A). On day 7 of hematopoietic differentiation (second row of Figure 5A), LNC000093 expression was increased by 5.98-fold and CD34, CXCR4, and GATA2 levels by 11.21-, 25.82-, and 56.63-fold, respectively (Figure 5C). On day 14 of hematopoietic differentiation (third row of Figure 5A), the above genes were more significantly upregulated relative to day 0, i.e., 33.93-fold (LNC000093), 41.94-fold (CD34), 57.29-fold (CXCR4), and 229.4-fold (GATA2) (Figure 5C).

A pair of sgRNAs was designed to recognize the target sequences (Supplementary Table 3) and allow Cas9 nuclease to cleave full-length LNC000093 genomic DNA (Supplementary Figure 5B). The effect of the deletion was validated via conventional PCR at the DNA level (Supplementary Figure 5C) and RT-qPCR at the transcript level. After CRISPR-Cas9-mediated deletion, LNC000093 was downregulated by 2.28-fold (Figure 5D; *P* < 0.05). Next, spontaneous differentiation was conducted for 3 or 7 days. All three differentiation markers (CD34, CXCR4, and GATA2) showed a significant reduction in increment (0.56- to 0.72-fold normalized to control) in LNC000093-CRISPR-deleted cells relative to the control group (Figure 5E; *P* < 0.01).

#### Bone Marrow Stromal Cell-Mediated Vascular Gene Expression Is Extrinsically Regulated by LNC000093-Bearing Exosomes

**FIGURE 6:**
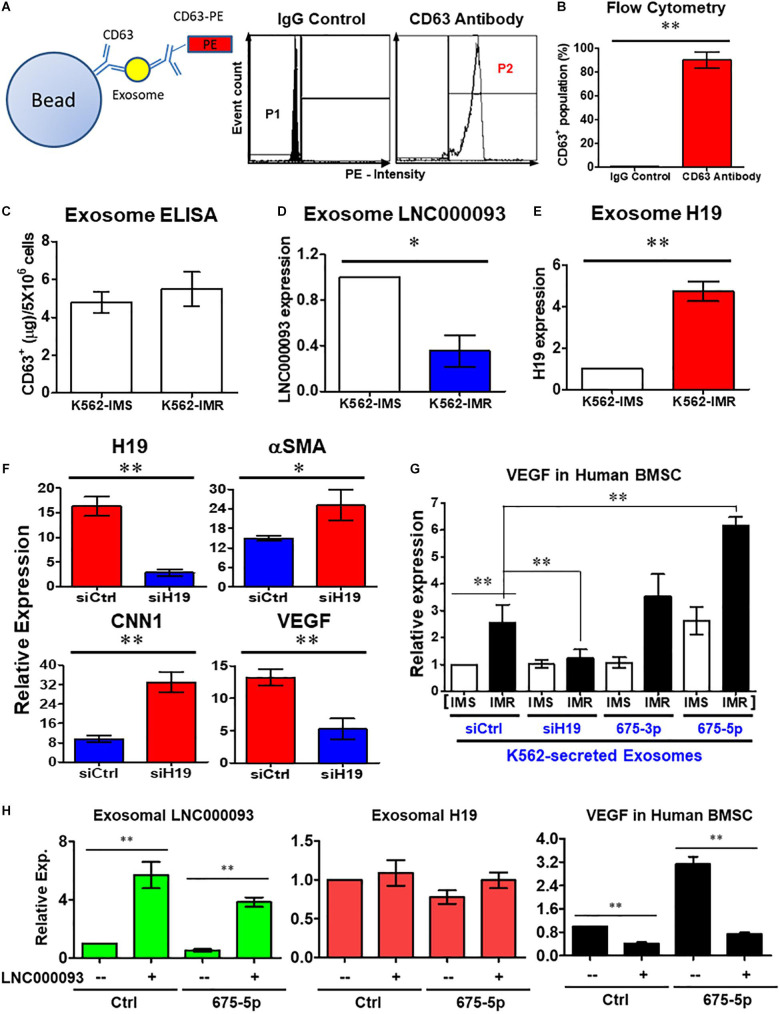
Extrinsic regulatory role of LNC000093 was revealed for bone marrow stromal cells. **(A,B)** Flow cytometry assay revealed CD63^+^-enriched exosomes were isolated from K562 with >80% purity (*n* = 3). The mean ± SD is shown for the results. ^∗∗^*P* < 0.01. **(C)** ELISA assay showed the total amount of isolated exosomes from K562-IMS and K562-IMR (*n* = 3). **(D,E)** The exosomal expression of LNC000093 **(D)** and H19 **(E)** in K562-IMS and K562-IMR was analyzed by RT-qPCR (*n* = 3). The mean ± SD is shown for the results. ^∗^*P* < 0.05, ^∗∗^*P* < 0.01. **(F)** RT-qPCR analysis of H19, aSMA, CNN1, and VEGF expression in BMSCs after siH19 transfection (*n* = 3). The mean ± SD is shown for the results. ^∗^*P* < 0.05, ^∗∗^*P* < 0.01. **(G)** RT-qPCR analysis showed VEGF expression in BMSCs after treating with exosomes isolated from K562-IMS and K562-IMR with or without transfection of siH19 or miR-675 mimics (*n* = 3). The mean ± SD is shown for the results. ^∗∗^*P* < 0.01. **(H)** RT-qPCR analysis showed the exosomal LNC000093 and H19 expression in LNC000093-overexpressed K562 with or without co-transfection of miR-675-5p mimics. A significant reduction of VEGF enhancement in BMSCs treated with LNC000093-overexpressed exosomes was revealed by RT-qPCR analysis (*n* = 3). The mean ± SD is shown for the results. ^∗∗^*P* < 0.01.

In addition to intrinsic regulation, extrinsic regulation of the bone marrow microenvironment plays an important role in CML progression (Kumar et al., 2020; Lefort and Maguer-Satta, 2020). Based on the findings of circulating lncRNAs, including H19 expression, we targeted the exosome compartment to investigate the potential regulatory role of LNC000093. Following exosome isolation and enrichment from K562, CD63^+^ populations with >80% purity were obtained (Figures 6A,B; *P* < 0.01). Using CD63-ELISA quantification, comparable total amounts of exosomes were determined between K562-IMS and IMR cells (Figure 6C). Both LNC000093 and H19 were detected in secreted exosomes and converse expression patterns observed in K562-IMR cells when compared to K562-IMS cells (Figures 6D,E; *P* < 0.05 and 0.01, respectively).

To establish the novel regulatory effects of the exosomal H19/miR-675-5p axis in the bone marrow microenvironment, synthetic oligonucleotide siRNA and miRNA mimics were transfected into both leukemic and bone marrow stromal cells (BMSCs). Following transfection of H19 siRNA (siH19) into human BMSCs, markers of vascular smooth muscle cells (αSMA and CNN1) were upregulated while the endothelial marker, vascular endothelial growth factor (VEGF), was downregulated (Figure 6F; *P* < 0.05 and 0.01, respectively). VEGF is a well-known factor that modulates angiogenesis within the tumor microenvironment and induces drug resistance status in leukemia (Zhang et al., 2006; Song et al., 2012). To investigate the potential interplay of secreted H19/miR-675-5p and LNC000093, and its effects on VEGF expression through exosomes, human BMSCs were co-cultivated with K562-secreted exosomes. Treatment with exosomes isolated from K562-IMR cells enhanced VEGF expression by 2.6-fold in human BMSCs when compared to exosomes isolated from K562-IMS cells (Figure 6G). Enhancement of VEGF expression was canceled out upon transfection of siH19 into K562 cells before harvesting of secreted exosomes, indicating that the regulatory effects are mainly exerted through the exosomal compartment. In contrast, treatment with exosomes from K562-IMR overexpressing miR-675-5p significantly enhanced VEGF expression by 6.2-fold (Figure 6G). This experimental setting has clearly demonstrated that upregulation of VEGF in human BMSCs is induced by H19/miR-675-5p-bearing exosomes secreted from leukemic cells, particularly with imatinib resistance.

The ‘competing effect’ derived from exosome LNC000093 was further addressed to determine its function in miR-675-5p-induced VEGF expression in human BMSCs. Expression of LNC000093 induced via plasmid transfection was detected in secreted exosomes isolated from either control miRNA or miR-675-5p-overexpressing K562 cells (left panel, Figure 6H). Expression of exosome H19 was not significantly altered by LNC000093 transfection (middle panel, Figure 6H). Significant inhibition of miR-675-5p-induced VEGF expression was achieved after treatment with K562-secreted exosomes overexpressing LNC000093 (right panel, Figure 6H). This inhibitory effect was also observed in the control miRNA group.

## Discussion

Over the past decade, lncRNAs have been extensively characterized in relation to different disease types, especially cancer and leukemia (Bhan et al., 2017; Wong et al., 2018; Gao et al., 2020). LncRNAs are highly versatile in terms of their mode of function and molecular targets, and remain a largely unexplored area. Recent research has reported the involvement of some lncRNAs in *BCR-ABL1*-positive CML and drug resistance, including H19, BGL-3 UCA1 and SNHG5 (Guo et al., 2014, 2015; He et al., 2017; Xiao et al., 2017). One of our lncRNA study targets, H19, is involved in BCR-ABL tumorigenesis. This lncRNA is highly expressed in CML and correlates with sensitivity to imatinib-induced apoptosis as well as *BCR-ABL1*-induced tumor growth (Guo et al., 2014). However, the potential linkage between H19 or its derived miR-675 and CML drug response has not yet been reported; hence H19/miR-675 have become the initial targets of our investigation in CML therapeutic resistance and subsequently led to the further exploration of their interaction with the newly identified lncRNA target.

**FIGURE 7:**
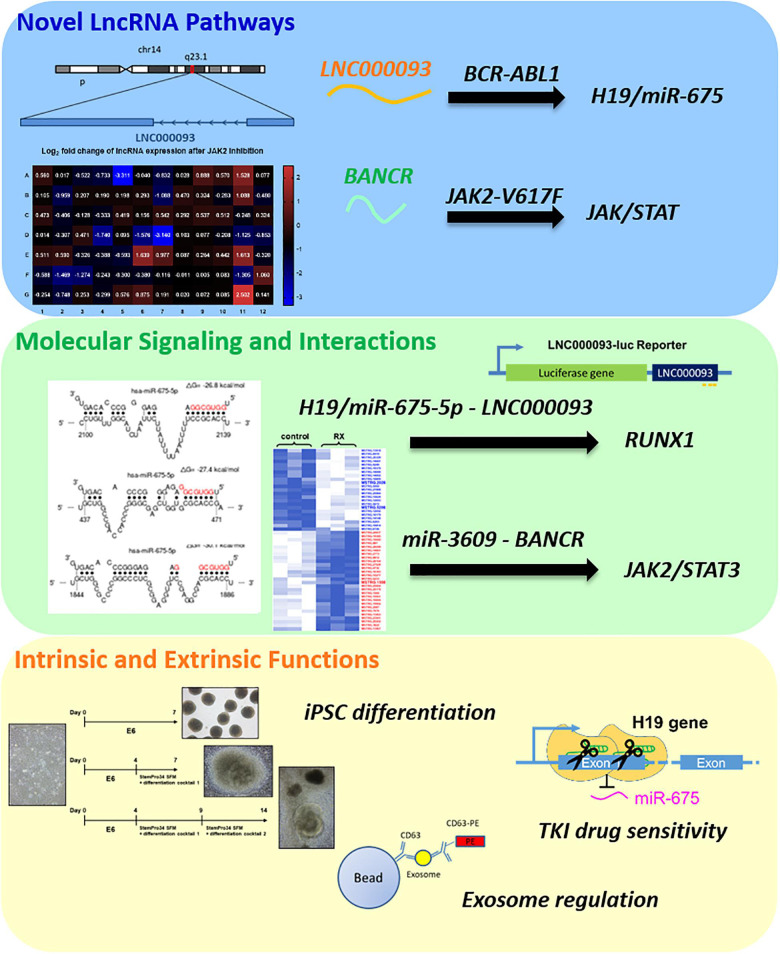
A summary of newly identified CML- and MPN-associated lncRNAs and their putative regulatory role.

In the present study, novel lncRNA pathways were identified in *BCR-ABL1*-positive CML and *BCR-ABL1*-negative MPN-derived cells (Figure 7). In imatinib-resistant (IMR) CML cells, both the known oncogene H19 and its derived miR-675 were upregulated (Figures 1C,D) while the novel lncRNA, LNC000093, was downregulated (Figure 1I). LNC000093 is a newly annotated lncRNA validated by our group and subsequent experiments revealed that its expression is regulated by H19/miR-675 through direct binding of miR-675-5p (Figure 3). RUNX1 expression in IMR cells was post-transcriptionally downregulated by miR-675-5p (Figure 4C). Mutations and translocation of *RUNX1* frequently occur in hematological malignancies and are proposed to underlie chemotherapeutic resistance (Sood et al., 2017). A recent study also unraveled a direct role of RUNX1 in response to cytotoxic drugs and subsequent apoptotic activities (Speidel et al., 2017). Our results demonstrated lower expression of RUNX1 in IMR cells (Figure 4B) corresponding to its potential role in drug response. Moreover, LNC000093 could act as a ceRNA to compete with RUNX1 for miR-675-5p to suppress H19/miR-675-5p-mediated RUNX1 inhibition, leading to de-repression of RUNX1 (Figure 4D) and enhanced sensitivity of response to TKI treatment. In other words, low expression of LNC000093 in IMR cells results in upregulation of miR-675-5p, which functions to suppress cell death upon imatinib treatment, leading to survival and non-response of CML cells to imatinib. The collective findings support a possible linkage between the LNC000093-H19/miR-675-RUNX1 axis and imatinib resistance in CML.

While RUNX1 has a potential regulatory role in drug response, it primarily serves as a crucial regulator of hematopoietic lineage by acting as a transcription factor (Imperato et al., 2015). Our results have shown that changes in LNC000093-mediated RUNX1 enhancement promote megakaryocytic differentiation induced by PMA (Figure 4F). We further investigated the regulatory effects of LNC000093 on other hematopoietic differentiation markers. LNC000093 was significantly increased during the differentiation process (Figure 5B), particularly when directed to hematopoietic lineage (Figure 5C), and its suppression (Figure 5D) led to reduction of early hematopoietic differentiation, as reflected by CD34, GATA2, and CXCR4 levels (Figure 5E). These findings suggest that LNC000093 has another physiological role in regulating differentiation toward hematopoietic lineages, which can be a possible cause of hematological disorders when it is dysfunctional.

Exosomal lncRNAs play emerging roles in the tumor microenvironment, including regulation of cancer cell migration, angiogenesis and immune response, which are related to the development of drug resistance (Wu et al., 2020). In addition to solid tumors, leukemia-secreted exosomes modulate the microenvironment in the bone marrow niche by altering the functions of mesenchymal stem and myeloid cells (Deng et al., 2020). However, the complex intercellular crosstalk mechanisms between leukemic and neighboring cells are yet to be understood. Therefore, it is essential to elucidate the regulatory mechanisms that are critical to modulation of CML drug response and the way in which leukemia-derived exosomes and potential exosomal lncRNAs influence the bone marrow microenvironment.

Our results have shown that H19/miR-675-5p- and LNC000093-bearing exosomes (Figures 6D,E) contribute to extrinsic regulation of bone marrow stromal cell-mediated vascular gene expression (Figures 6F,G), which may in turn alter the bone marrow microenvironment. Treatment of BMSCs with exosomes isolated from IMR cells resulted in enhancement of VEGF expression that was further augmented with overexpression of miR-675-5p (Figure 6G). Meanwhile, increased exosomal LNC000093 led to diminished miR-675-5p-induced VEGF expression (right panel, Figure 6H). Our results have demonstrated that H19/miR-675 and LNC000093 from leukemic cells are transported through extracellular vesicle exosomes and exert effects on BMSCs to alter the expression of VEGF, which is a representative promoter of tumor angiogenesis and is associated drug resistance (Fan et al., 2012; Finley and Popel, 2013).

In addition to investigating lncRNAs in *BCR-ABL1*-positive CML, we also put efforts to explore any lncRNAs being involved in *BCR-ABL1*-negative MPN since very limited research has been done on it. Initial screening with PCR array led to the identification of a number of lncRNAs showing significant differential expression upon JAK2 inhibition in *JAK2*-V617F^+^ MPN model cell HEL 92.1.7. Among these, BANCR was the most significantly downregulated target, showing dose-dependent expression changes (Figure 2E). Notably, ruxolitinib treatment of K562 cells, which is wild-type for JAK2, did not cause any expression change of BANCR, which indicates the high specificity of BANCR regulation in mutant *JAK2*-V617F signaling (Figure 2F). BANCR was upregulated via ectopic expression of *JAK2*-V617F (Figure 2G), supporting its identification as a downstream target of the *JAK2*-V617F-mediated signaling pathway. Additional studies are warranted to further elucidate the role of BANCR in *JAK2*-V617F signaling and related pathogenesis. However, such findings, together with converse expression pattern of H19 in *BCR-ABL1*-pos and *BCR-ABL1*-neg MPNs upon TKI treatment (Supplementary Figures 1A, 4B) highlights the specificity of lncRNA regulation mechanisms in different oncogenic pathways.

In addition, we aimed to identify novel lncRNAs and search for new regulatory networks with the aid of next-generation sequencing and bioinformatics tools to expand our knowledge. Accumulating evidence has shown that lncRNAs are involved in competitive regulatory interactions and act as ceRNAs, which sequester miRNAs and diminish their suppressive effects on other transcripts (Chan and Tay, 2018). Given that transcripts containing specific miRNA response elements could serve as ceRNAs and post-transcriptionally regulate gene expression (Qi et al., 2015), we performed *in silico* analysis to identify putative ceRNAs for BANCR from our newly identified *JAK2*-associated novel lncRNAs. Our results have shown that 8 novel lncRNAs possess shared binding potential for miR-3609 with BANCR and some JAK/STAT pathway associated genes (Supplementary Tables 7, 8), generating an inter-regulatory network. We further investigated the correlation between the locations of differentially expressed novel lncRNAs from our RNA-seq and several MPN-related CNVs. MSTRG.2026 and MSTRG.1558 were novel lncRNAs showing significant differential expression in our RNA-seq data (Supplementary Table 5) and they are located within two CNVs with gain and loss of copy numbers in MPN patients, respectively (Supplementary Table 6). It is expected that their expression levels would be aberrant according to the altered copy number and hence lead to deregulatory effects that ultimately enhance disease progression. An example lncRNA, SNHG6, has been shown to be highly expressed in colorectal cancer tissues and cells as a result of DNA copy number gains due to frequent amplification of this genomic region in colorectal cancer (Xu et al., 2019). Subsequent experiments have demonstrated that SNHG6 promotes growth and metastasis of colorectal cancer, and its upregulation is associated with tumor progression and poor prognosis. Thus, CNV analysis could provide insights into clinical relevance for potential diagnosis and therapy. Indeed, the *BCR-ABL1*-neg MPN patient samples we used for CNV analysis are all from primary myelofibrosis subtype, which is the more severe subtype and has the highest risk of leukemic transformation (Yogarajah and Tefferi, 2017). Therefore, the identified CNVs and their influence on lncRNA expression could implicate certain clinical significance for MPN progression that are worthy of deeper examination.

In summary, we have shown that the increased H19/miR-675-5p levels contribute to imatinib resistance in CML cells through regulation of RUNX1 expression, and newly identified LNC000093 serves as a ceRNA for miR-675-5p with functionally significant effects. The extrinsic regulatory role of exosomal H19/miR-675 and LNC000093 in the bone marrow microenvironment is also demonstrated, suggesting another remarkable route of developing drug resistance. High-throughput profiling of *JAK2*-mutant MPN model cells screened out a number of known and novel lncRNA targets that are highly associated with *JAK2*-V617F signaling pathway and deserve further investigation. Altogether, we have explored the molecular basis of MPNs in different aspects mainly through *in vitro* models with distinct genetic characteristics of *BCR-ABL1*-positive or -negative MPNs in the current study, but we prospect for future translational research studies in the same field with compelling clinical relevance so that potential lncRNA biomarkers could be established for MPNs. Besides, our data highlight the comprehensive crosstalk between lncRNAs and their versatile molecular targets and contribute to the mechanistic insights of lncRNA-miRNA-mRNA axes in hematological malignancies.

## Data Availability Statement

All data generated and analyzed in this study are included in this article and its Supplementary Material. The datasets of RNA-seq generated for this study can be found in online repositories. The RNA-seq raw data can be found in SRA with accession number PRJNA703413. The novel lncRNA LNC000093 sequence can be found in GenBank with accession number MW387002.

## Ethics Statement

The studies involving human participants were reviewed and approved by Human Subjects Ethics Sub-committee, The Hong Kong Polytechnic University. The patients/participants provided their written informed consent to participate in this study.

## Author Contributions

C-LH and SY conceived and designed the experiments. C-LH and NW drafted the manuscript. C-LH, NW, and SL prepared the figures and tables. EC and HM coordinated the collection of patient samples and the demographic data. NW, FM, AA, and JS performed the experiments. SL and JS performed the bioinformatics analysis. WJ and W-CL supervised the involved students and staff, and provided useful advice. C-LH and SY critically evaluated and revised the manuscript. All authors read and approved the final manuscript.

## Conflict of Interest

The authors declare that the research was conducted in the absence of any commercial or financial relationships that could be construed as a potential conflict of interest.

## Publisher’s Note

All claims expressed in this article are solely those of the authors and do not necessarily represent those of their affiliated organizations, or those of the publisher, the editors and the reviewers. Any product that may be evaluated in this article, or claim that may be made by its manufacturer, is not guaranteed or endorsed by the publisher.
