# Supplementary material for: The Tyrosine Kinase-Driven Networks of Novel Long Non-coding RNAs and Their Molecular Targets in Myeloproliferative Neoplasms
Source: Front Cell Dev Biol. 2021 Aug 3;9:643043. doi: 10.3389/fcell.2021.643043 (PMC8369571; doi:10.3389/fcell.2021.643043)
Supplement: Supplementary file 1 [file Data_Sheet_1.PDF]

Supplementary Material

Supplementary Table 1. Information of specimens collected from MPN patients.

CML patients	(*BCR-ABL1*-positive)		
Sample No.	WBC counts (10^9/L)	BCR-ABL1 transcript type	Interpretation
**465**	7.70	e14a2	Check drug compliance and repeat RT-qPCR later
**688**	7.97	e13a2	Check drug compliance and repeat RT-qPCR later
**690**	7.97	e13a2	Check drug compliance and repeat RT-qPCR later
**700**	-	e14a2	> or = 5 log reduction
**712**	5.40	e13a2	N.A.
**804**	9.80	e14a2	> or = 5 log reduction
**808**	15.2	e14a2	3 log reduction
**848**	4.98	e14a2	> or = 5 log reduction
**881**	4.36	e14a2	> or = 5 log reduction
**886**	6.25	e14a2	Check drug compliance and repeat RT-qPCR later

MPN patients	(*BCR-ABL1*-negative)				
Sample No.	WBC (10^9/L)	RBC (10^{12}/L)	PLT (10^9/L)	Mutation type	Interpretation
**008**	9.84	5.12	45.5	JAK2 V617F	PMF/PV
**012**	21.8	2.32	57.2	-	PMF
**014**	7.21	2.68	713	CALR 52del (type 1)	PMF
**027**	32.7	2.92	506	CALR 52del (type 1)	PMF
**118**	4.97	2.77	200	CALR 39del	PMF
**125**	7.44	3.84	129	JAK2 V617F	PMF
**126**	19.3	3.28	298	CALR 52del + point mutation	PMF
**144**	7.71	2.76	84.1	JAK2 V617F	PMF

Supplementary Table 2. List of primers used in the study.

Primer sequences (5' → 3')		
qPCR primer		
MDR1	Forward	ATG CTA TAA TGC GAC AGG AG
	Reverse	CCC AGT GAA AAA TGT TGC CA
H19	Forward	ATC GGT GCC TCA GCG TTC G
	Reverse	CTC TGT CCT CGC CGT CAC A
LNC000093	Forward	GCA TTA CCT CTA TCC TAC CTG G
	Reverse	GGT TGT GTT TTA CTC CTC GCA GA
LNC000114	Forward	ATG TGG GGA GGG GCA AAG AAA T
	Reverse	TGG CAG CAC TAA GCA AAG GC
BANCR	Forward	TGA GCC TCT ATT GGA ATC AGC
	Reverse	GCC AGG GAT GAC TTG CGT ATA
CD34	Forward	CCA CAA CAA ACA TCA CAG AAA CGA
	Reverse	GGT GGT GAA CAC TGT GCT GAT TA
CXCR4	Forward	TGC CCT CCT GCT GAC TAT TC
	Reverse	CCA ACC ATG ATG TGC TGA AAC TG
GATA2	Forward	GAA CCG ACC ACT CAT CAA GC
	Reverse	CGT CTG ACA ATT TGC ACA ACA GG
GAPDH	Forward	AGG TCG GAG TCA ACG GAT TTG
	Reverse	TGA AGG GGT CAT TGA TGG CAA CA
PCR primer		
H19-CRISPR checking	Forward	GGG CCA CCC CAG TTA GAA AA
	Reverse	GTC CTG CTT GTC ACG TCC AC
LNC000093-CRISPR checking	Forward	ATG TTG GTG TAT CTT GAG ATC CTC
	Reverse	TCC CCA GTT GTA CTC CAT CTG T

Supplementary Table 3. **List of antibodies and supplement for iPSC differentiation.**

Name	Species	Company	Cat. No.
**Antibodies**			
RUNX1	Mouse	Santa Cruz Biotechnology	sc-365644
β-actin	Mouse	Santa Cruz Biotechnology	sc-47778
JAK2	Rabbit	Cell Signaling Technology	3230
Phospho-STAT3	Rabbit	Cell Signaling Technology	9131
Phospho-STAT5	Rabbit	Cell Signaling Technology	9359
STAT3	Rabbit	Santa Cruz Biotechnology	sc-482
STAT5	Mouse	Santa Cruz Biotechnology	sc-74442
Anti-rabbit IgG, HRP-linked	Rabbit	Cell Signaling Technology	7074
Anti-mouse IgG, HRP-linked	Mouse	Cell Signaling Technology	7076
**iPSC differentiation supplement**			
VEGF		PeproTech	100-20A
SCF		PeproTech	300-07
Flt3-L		PeproTech	300-19
IL-3		PeproTech	200-03
IL-11		PeproTech	200-11
EPO		PeproTech	100-64
IGF-1		PeproTech	100-11
BMP4		Prospec	CYT-361
TPO		Prospec	CYT-302
Transferrin		Prospec	PRO-315
Ascorbic acid		Sigma	A8960
1-thioglycerol		Sigma	M1753
b-FGF		PeproTech	100-18B
Polyvinyl alcohol (PVA)		Sigma	P8136

Supplementary Table 4. SgRNA for CRISPR-cas9 system.

Target	sgRNA sequences (5' → 3')	PAM site
H19 sgRNA1	GCTAGGACCGAGGAGCAGGGTG	AGG
H19 sgRNA2	GATCGGTGCCTCAGCGTTCGGGC	TGG
LNC000093 sgRNA1	TCCCAACTCCCACGTTAGAG	TGG
LNC000093 sgRNA2	ACATTTTTGCTTAAGAACTT	CGG

Supplementary Table 5. Top 20 up- and down-regulated lncRNA in HEL after Ruxolitinib treatment.

Gene ID†	Gene	baseMean††	Log_2 Fold Change	lfcSE†††	p-value	p_{adj}
ENSG00000274370	AC144831.3	19.698287	2.914185	0.758311	0.000122	0.000693
ENSG00000244382	RP11-373I8.1	39.944185	2.474340	0.449706	3.75E-08	4.42E-07
ENSG00000240050	RP1-93H18.1	14.628234	2.257259	0.802638	0.004919	0.01781
ENSG00000223727	AC026188.1	13.691442	2.002816	0.711658	0.004888	0.017725
ENSG00000275527	CTD-3154N5.2	13.778523	1.996198	0.740527	0.007025	0.024195
ENSG00000278921	EPB41L4A-AS2	15.053160	1.929660	0.687477	0.005003	0.018077
ENSG00000261888	AC144831.1	25.308926	1.823062	0.590087	0.002005	0.008225
MSTRG.19902	MSTRG.19902	33.419314	1.790756	0.476815	0.000173	0.000951
MSTRG.4736	MSTRG.4736	2665.882866	1.757615	0.114254	2.12E-53	6.87E-51
ENSG00000266651	RP11-138I1.3	39.525033	1.727911	0.453195	0.000137	0.000774
MSTRG.25004	MSTRG.25004	338.792323	1.697930	0.265227	1.54E-10	2.66E-09
ENSG00000232470	RP11-313D6.3	49.670179	1.680342	0.429571	9.17E-05	0.000541
MSTRG.1558	MSTRG.1558	19.756380	1.629320	0.627209	0.009384	0.030864
MSTRG.20202	MSTRG.20202	894.430509	1.621538	0.127750	6.46E-37	9.16E-35
ENSG00000251434	RP11-315A17.1	27.901942	1.617880	0.592863	0.006354	0.022195
ENSG00000234690	AC073283.4	23.228258	1.609461	0.572161	0.004909	0.017779
ENSG00000231439	WASIR2	85.249281	1.593935	0.294263	6.07E-08	6.93E-07
ENSG00000258084	RP11-754N21.1	25.564300	1.559774	0.537576	0.003714	0.013999
ENSG00000232855	AF131217.1	18.738069	1.548156	0.629797	0.013964	0.043218
ENSG00000225173	XXbac-BPG308K3.5	28.007982	1.489438	0.539012	0.005722	0.020299
MSTRG.5206	MSTRG.5206	140.924638	-5.270468	0.388686	6.94E-42	1.34E-39
MSTRG.12693	MSTRG.12693	318.926452	-3.320190	0.237110	1.50E-44	3.42E-42
MSTRG.16179	MSTRG.16179	34.214206	-3.030161	0.522689	6.74E-09	8.93E-08
ENSG00000238062	SPATA3-AS1	11.359000	-2.713517	0.907172	0.002779	0.010914
MSTRG.5060	MSTRG.5060	45.454877	-2.680041	0.422096	2.16E-10	3.67E-09
MSTRG.14148	MSTRG.14148	153.166073	-2.641340	0.268349	7.35E-23	4.94E-21
MSTRG.25984	MSTRG.25984	242.337475	-2.514937	0.224062	3.10E-29	3.14E-27
MSTRG.14000	MSTRG.14000	173.362803	-2.484996	0.313933	2.46E-15	7.82E-14
MSTRG.14001	MSTRG.14001	23.901599	-2.435222	0.632455	0.000118	0.000676
MSTRG.14002	MSTRG.14002	26.118020	-2.431415	0.578305	2.62E-05	0.000176
ENSG00000274021	RP11-823E8.3	16.776498	-2.389231	0.719458	0.000897	0.004073
ENSG00000228340	MIR646HG	27.464887	-2.335150	0.564576	3.53E-05	0.000229
ENSG00000266378	RP11-214O1.3	11.772855	-2.253249	0.818848	0.005928	0.020936
ENSG00000266709	RP11-214O1.2	57.558749	-2.130532	0.402905	1.24E-07	1.34E-06
ENSG00000261685	RP11-401P9.4	24.502471	-2.047620	0.554804	0.000224	0.0012
MSTRG.13918	MSTRG.13918	24.637600	-2.046617	0.635149	0.001272	0.005505
MSTRG.9249	MSTRG.9249	13.452298	-1.986449	0.753081	0.008345	0.027992
MSTRG.2026	MSTRG.2026	25.312443	-1.984915	0.537325	0.000221	0.001186

**MSTRG.6203**	MSTRG.6203	90.275618	-1.984434	0.398111	6.21E-07	5.87E-06
**MSTRG.21888**	MSTRG.21888	30.217200	-1.983776	0.497453	6.67E-05	0.000407

† Gene ID starting with MSTRG are newly identified novel lncRNA from the RNA-seq data
†† the average of the normalized counts taken over all samples
††† standard error of the log_2 fold change estimate

Supplementary Table 6. Location of MPN-related CNV and differentially expressed lncRNAs.

Genomic coordinate	lncRNA	lncRNA type	MPN sample	Copy number
**chrX:45745211-45770274**	MIR222HG	Known	MPN012	1
**chrX:45745211-45770274**	MIR222HG	Known	MPN027	1
**chrX:45745211-45770274**	MIR222HG	Known	MPN126	1
**chrX:45764772-45765299**	RP6-99M1.3	Known	MPN012	1
**chrX:45764772-45765299**	RP6-99M1.3	Known	MPN027	1
**chrX:45764772-45765299**	RP6-99M1.3	Known	MPN126	1
**chrX:71697196-71706455**	LINC00891	Known	MPN012	0
**chrX:71697196-71706455**	LINC00891	Known	MPN027	1
**chrX:71697196-71706455**	LINC00891	Known	MPN126	1
**chrX:71771506-71784726**	MSTRG.26599	Novel	MPN012	0
**chrX:71771506-71784726**	MSTRG.26599	Novel	MPN027	1
**chrX:71771506-71784726**	MSTRG.26599	Novel	MPN126	1
**chr1:124804374-124817081**	MSTRG.1558	Novel	MPN014	1
**chr1:124804374-124817081**	MSTRG.1558	Novel	MPN126	1
**chr1:124804374-124817081**	MSTRG.1558	Novel	MPN144	1
**chr1:124908709-124912327**	MSTRG.1560	Novel	MPN014	1
**chr1:124908709-124912327**	MSTRG.1560	Novel	MPN126	1
**chr1:124908709-124912327**	MSTRG.1560	Novel	MPN144	1
**chr1:161450633-161458545**	MSTRG.2026	Novel	MPN118	4
**chr1:167863281-167890957**	MSTRG.2087	Novel	MPN118	4
**chr13:50108816-50116015**	MSTRG.7076	Novel	MPN126	1
**chr20:34098756-34101495**	MSTRG.16383	Novel	MPN125	1
**chr20:44210960-44226027**	OSER1-AS1	Known	MPN125	1

Supplementary Table 7. Potential binding region of miR-3609 on BANCR and novel lncRNAs.

lncRNA	Transcript variant	Binding region	Seed sequence position	ΔG* (kcal/mol)
**BANCR**		22-36	30-36	-17.5
**MSTRG.5060**		1664-1688	1681-1687	-21.9
**MSTRG.5206**	Variant 1	1639-1679	1673-1678	-26.5
		90-107	102-107	-16.1
	Variant 3	1541-1581	1575-1580	-26.5
		90-107	102-107	-16.1
**MSTRG.12693**		1674-1702	1696-1702	-17.2
		292-303	297-302	-14.1
**MSTRG.13918**		8-28	22-27	-22.1
**MSTRG.14000**	Variant 1	3-43	37-42	-26.0
	Variant 2	3-43	37-42	-26.0
	Variant 3	3-43	37-42	-26.0
	Variant 4	3-43	37-42	-26.0
**MSTRG.14001**		1-35	29-34	-23.8
**MSTRG.14002**		1-35	29-34	-23.8
**MSTRG.14148**		1443-1483	1477-1483	-16.3
		771-795	789-794	-18.0
		2488-2508	2502-2507	-25.3

*ΔG = A measure of stability for miRNA:target hybrid as computed by RNAhybrid. The more negative the value, the more stable the binding.

Supplementary Table 8. Potential binding region of miR-3609 on JAK/STAT related-genes.

Genes	Transcript variant	Binding region	Seed sequence position	ΔG* (kcal/mol)
**IL6ST**	variant 1	728-762	756-762	-14.5
		4792-4809	4804-4809	-14.8
		4020-4046	4041-4046	-15.4
		2090-2106	2100-2106	-16.7
		4603-4651	4646-4651	-14
**IL12A**	variant 1	1199-1220	1214-1220	-21.8
**CNTF**		1356-1376	1370-1376	-21.3
		1695-1711	1705-1711	-20.7
**CCND1**		1592-1634	1628-1634	-21.5
		2008-2066	2061-2066	-13.2
**SPRY3**	variant 1	3430-3452	3446-3452	-16.7
		8065-8090	8084-8090	-15.3
**STAM2**		4454-4474	4468-4474	-14.5
		2200-2220	2215-2220	-19.3
		2289-2315	2309-2315	-20
		4815-4855	4849-4854	-27
**SOCS4**	variant 1	1842-1854	1848-1853	-13.8
		1144-1168	1162-1167	-15
		6528-6565	6559-6565	-15.6
		6773-6794	6789-6794	-14.1
**JAK2**	variant 1	2115-2134	2129-2134	-18.4
		6269-6296	6290-6296	-21.6
		5105-5121	5116-5121	-20.1
		6041-6072	6066-6072	-17.8
		2679-2696	2690-2695	-15.1
**PIAS1**	variant 1	3545-3570	3565-3570	-17.3
**LIFR**	variant 1	1419-1444	1439-1444	-19.2
		10006-10031	10026-10031	-13.6

*ΔG = A measure of stability for miRNA:target hybrid as computed by RNAhybrid. The more negative the value, the more stable the binding.

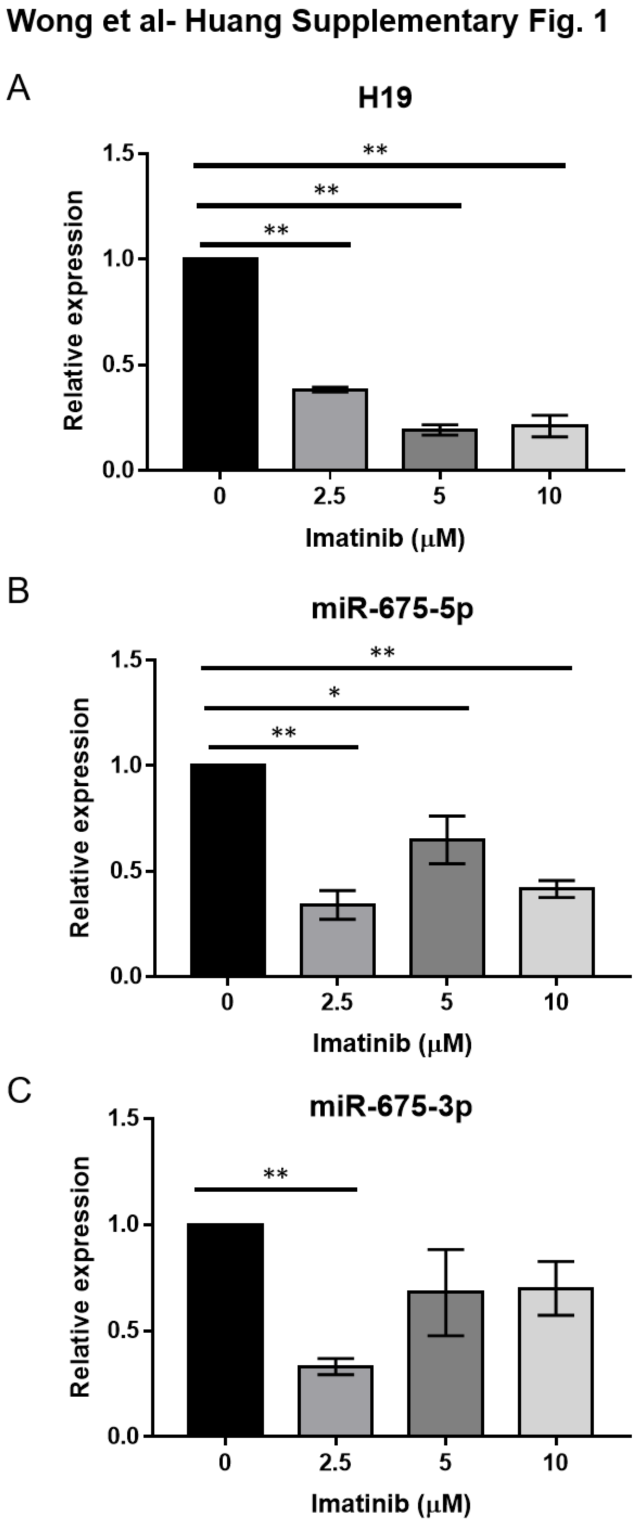

Supplementary Figure 1. **A-C** RT-qPCR analysis of H19 (**A**), miR-675-5p (**B**) and miR-675-3p (**C**) expression in K562 after imatinib treatment with different dosage. The mean ± SD is shown for RT-qPCR results. *$P < 0.05$; **$P < 0.01$.

Wong et al- Huang Supplementary Fig. 2

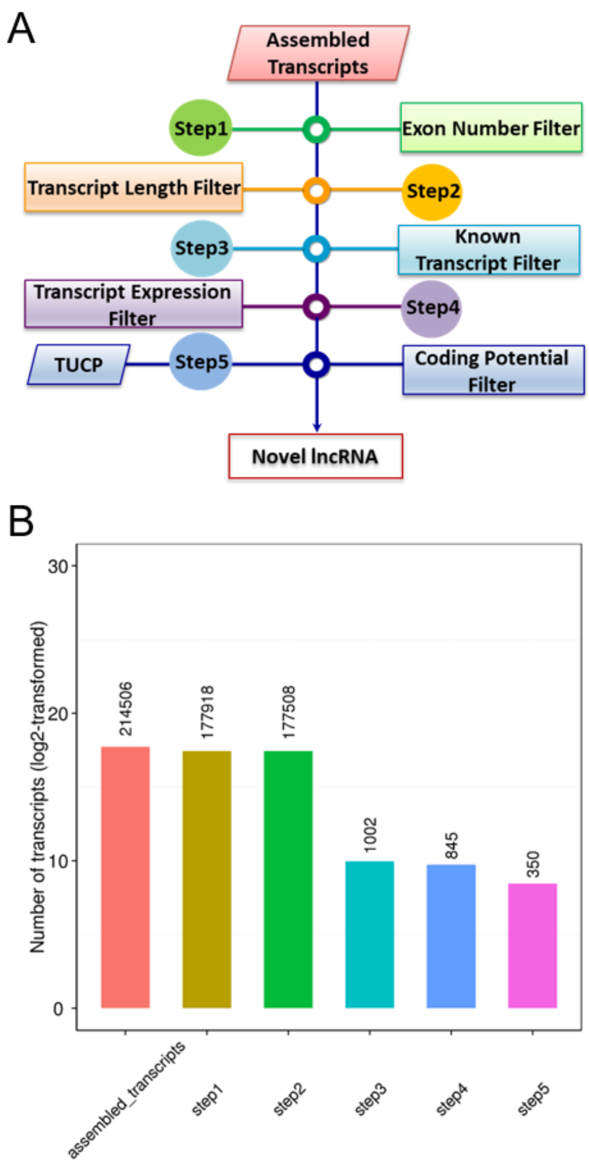

Supplementary Figure 2. (**A**) The workflow of novel lncRNA prediction and filtering. (**B**) Statistics of lncRNA filtering. The bar chart showed the number of transcripts after filtering out in each step.

Wong et al- Huang Supplementary Fig. 3

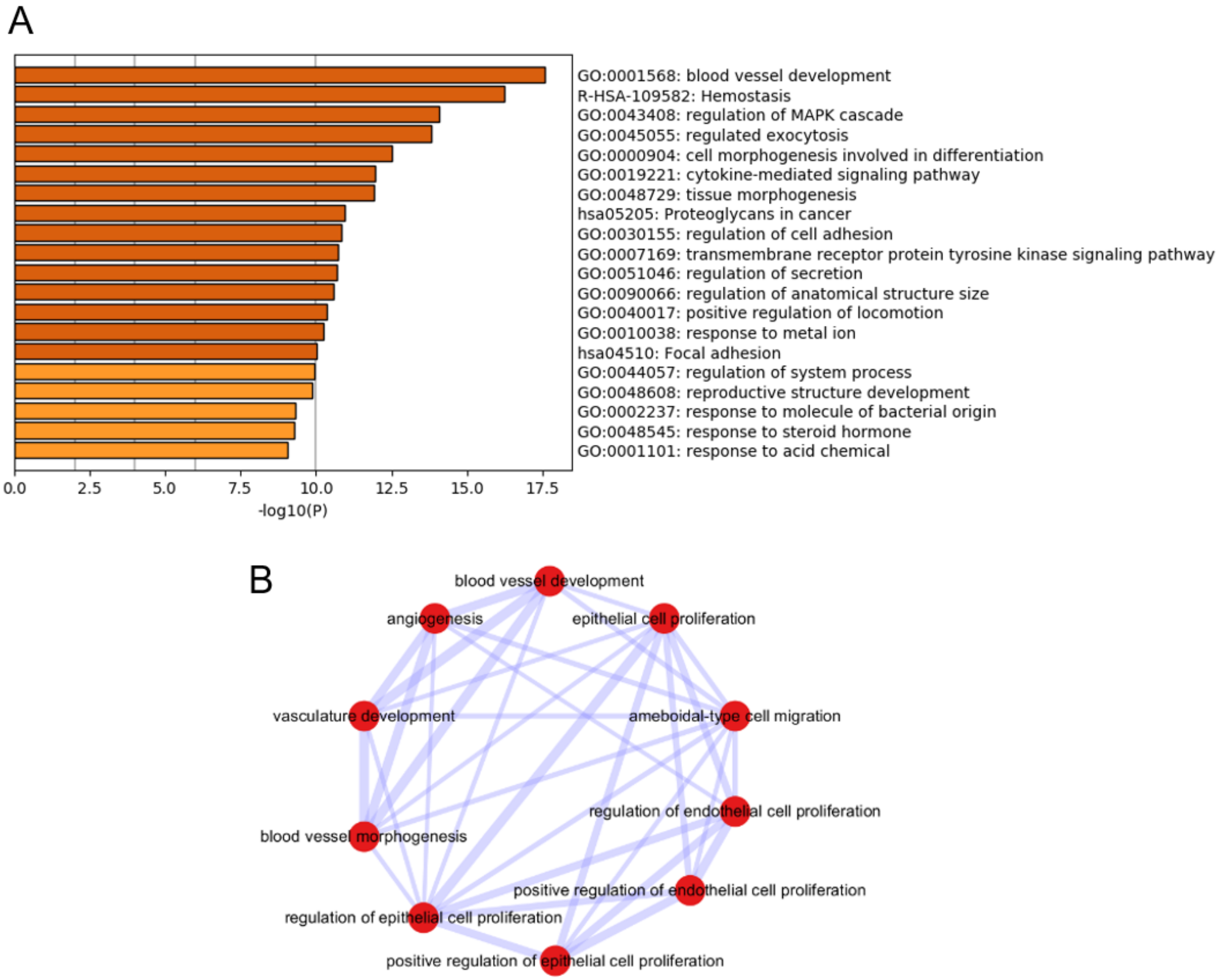

Supplementary Figure 3. (**A**) The bar graph showed top Metascape enriched terms across differentially expressed genes and is colored according to *p*-values. (**B**) Gene sharing network of sub-terms under the top enriched blood vessel development term. The thickness of lines positively correlated to amount of sharing genes.

Wong et al- Huang Supplementary Fig. 4

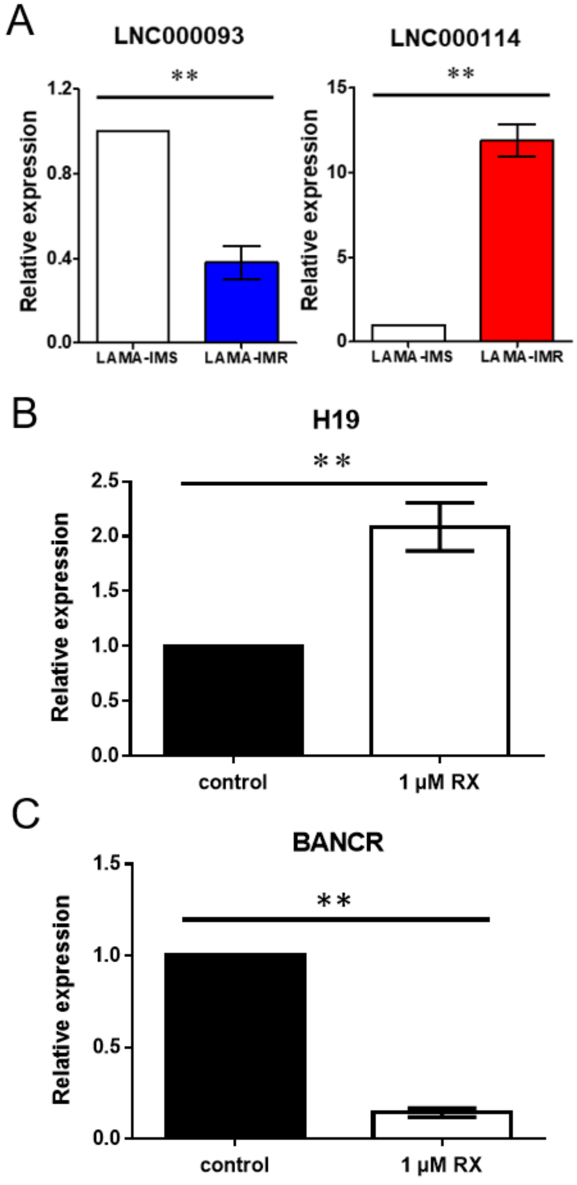

Supplementary Figure 4. (**A**) RT-qPCR analysis revealed a significant downregulation of LNC000093 and upregulation of LNC000114 in LAMA-84-IMR relative to LAMA-84-IMS (n = 4). (**B**) RT-qPCR analysis detected an upregulation of H19 in HEL upon ruxolitinib treatment (n = 5). (**C**) RT-qPCR analysis revealed a significant downregulation of BANCR in SET-2 upon ruxolitinib treatment (n = 4). The mean ± SD is shown for RT-qPCR results. ***$P < 0.01$.

Wong et al- Huang Supplementary Fig. 5

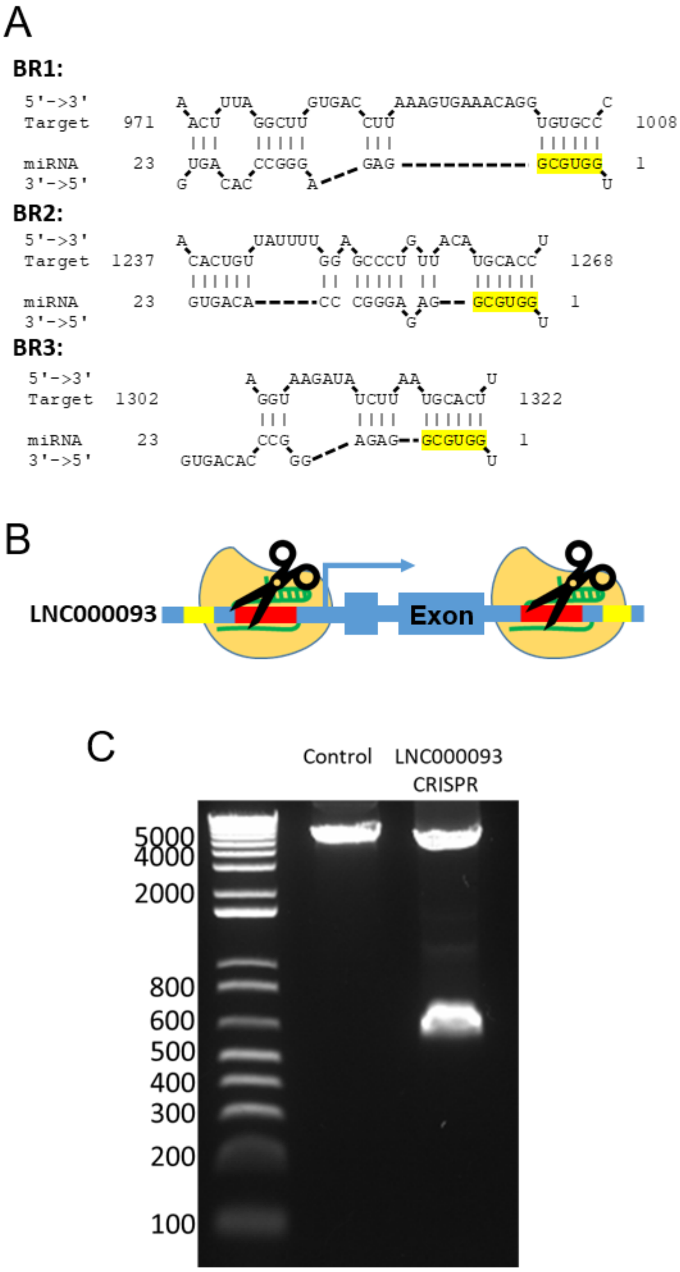

Supplementary Figure 5. (**A**) LNC000093 contains three putative binding regions (BRs) for miR-675-5p with the seed sequence (GGUGCG). (**B**) A schematic diagram showed Cas9 enzymes are guided by sgRNAs to their target regions (red) and generate cleavage to delete the full-length of LNC000093 genomic region. A pair of primers flanking the LNC000093 (yellow) are designed to confirm the deletion by PCR. (**C**) Gel electrophoresis showed an extra band for the LNC000093-CRISPR sample, indicating a successful deletion. The parental amplicon size is 5kb and LNC000093-deletion would lead to a shorter amplicon size with ~560 bp.
